# Absorption Properties of Large Complex Molecular Systems: The DFTB/Fluctuating Charge Approach

**DOI:** 10.1021/acs.jctc.1c01066

**Published:** 2022-02-20

**Authors:** Piero Lafiosca, Sara Gómez, Tommaso Giovannini, Chiara Cappelli

**Affiliations:** Scuola Normale Superiore, Classe di Scienze, Piazza dei Cavalieri 7, 56126 Pisa, Italy

## Abstract

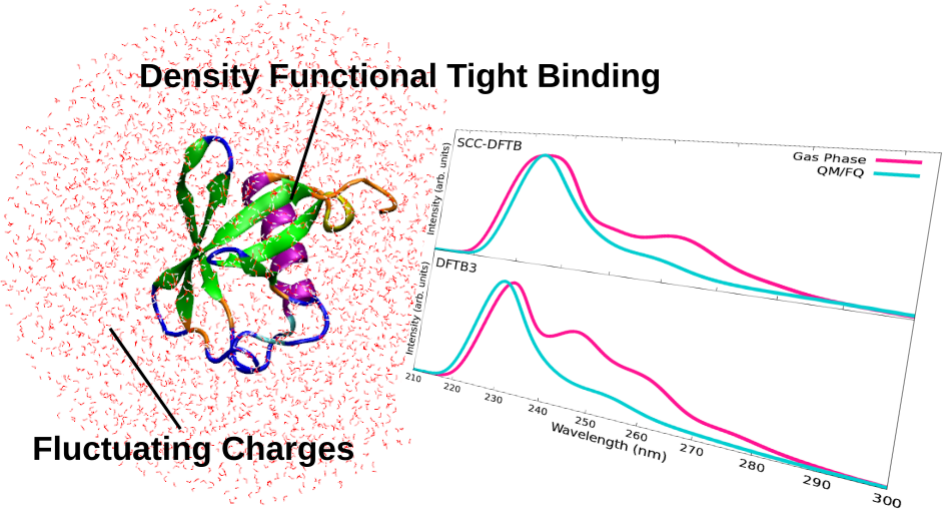

We report on the first formulation of a novel polarizable QM/MM approach, where the density functional tight binding (DFTB) is coupled to the fluctuating charge (FQ) force field. The resulting method (DFTB/FQ) is then extended to the linear response within the TD-DFTB framework and challenged to study absorption spectra of large condensed-phase systems.

## 1 Introduction

The theoretical modeling of large molecular systems, with application in biological and technological fields, is one of the most challenging tasks for theoretical and computational chemistry.^1,2^ In fact, the description of large molecular systems requires the treatment of a large number of degrees of freedom, from both nuclear and electronic points of view.^1,2^ For this reason, high-level quantum mechanics (QM) methods are usually not applicable because they are usually associated with an unfavorable scaling with the number of atoms (and electrons).^3^ Different strategies, usually based on chemical intuition, can be exploited to reduce the dimensionality of the system and to make high-level QM approaches applicable.^4−9^ This is, for instance, the case for local excitations, which take place in a specific part of the considered molecule.^5,8,10^ However, in many cases, especially for biomolecules, such an approximation may not be chemically justified because the phenomenon is the result of changes in the whole structure.

Semiempirical QM methods have been developed to treat in a realistic way this kind of system, which can be constituted by thousands of atoms.^11−14^ Such methodologies introduce a set of integral approximations and parametrizations that make the computation particularly cheap. Clearly, the accuracy of each approach strongly depends on the quality of the parametrization. Among semiempirical methods, one of the most used is the density functional tight binding (DFTB) approach.^15−17^ The theoretical starting point of such method is the density functional theory (DFT) energy in the Kohn–Sham (KS) framework, expressed by means of a linear combination of atomic orbitals (LCAO) over a minimal basis set. This quantity is then approximated by means of a Taylor expansion with respect to a reference density truncated at different orders by generating a hierarchy of DFTB methods.^15^ In particular, the self-consistent charge DFTB approach (SCC-DFTB), which corresponds to a second-order expression of the KS energy, has been successfully applied to the calculation of energies, geometries, and vibrational frequencies of small organic molecules; its accuracy when compared with experimental values is comparable to that of full DFT calculations performed with a double-ζ plus polarization basis set.^17^ Moreover, a time-dependent DFTB (TD-DFTB) approach has been developed to calculate excitation energies in a tight-binding fashion.^18,19^ However, it has been shown that the standard pure or hybrid DFT functionals are not able to accurately treat charge-transfer excitations because their extension to the corresponding long-range-corrected versions is necessary.^20,21^ In this context, the time-dependent long-range-corrected DFTB approach (TD-LC-DFTB)^22^ has recently been proposed and explicitly designed for the treatment of charge-transfer states in large chromophores.

The DFTB approximation allows the boundaries of the systems treatable by most *ab initio* approaches to be pushed, making possible the QM description of large biomolecules, such as proteins.^19,23^ However, most biomolecules are typically dissolved in an external environment, as water is the most common physiological solvent.^24^ As for small organic molecules, also in such cases, the external aqueous solution may strongly affect the properties of the biological system.^25^ To take into account the solvent effect provided by water, the best compromise between computational cost and accuracy is to resort to the so-called focused models, in which the target system and the environment are described at different levels of theory based on the assumption that the phenomenon is carried on by the target and the environment just perturbs it.^26−28^ Among the different focused models, the most accurate are the polarizable QM/molecular mechanics approaches.^29−44^ In such methods, the environment molecules are classically and atomistically described by means of a polarizable force field, and mutual solute–solvent polarization is taken into account. In particular, excellent performances have been reported for the QM/fluctuating charge (QM/FQ) in the description of aqueous solutions^45^ and recently for different solvents.^46^ In such an approach, each solvent atom is endowed with a charge that is adjusted to the external potential generated by the solute density.^28,45,47^ Such charges then polarize the QM density by entering the QM Hamiltonian in a mutual polarization fashion. In its basic formulation, the QM/FQ interaction is limited to electrostatics; however, nonelectrostatic interactions can also be considered.^48^

In this work, we have substantially extended the applicability of the polarizable QM/FQ approach by proposing a novel polarizable QM/FQ scheme based on the DFTB approach for the QM portion, allowing for the treatment of large, complex biomolecular systems. To the best of our knowledge, this is the first time that DFTB has been coupled to a polarizable MM approach. The newly developed DFTB/FQ approach has also been extended to the linear response regime by means of the time-dependent DFTB (TD-DFTB) approximation,^18,19^ and it has been tested to reproduce the excitation energies of doxorubicin (DOX), an anticancer drug, in aqueous solution and intercalated in DNA and ubiquitin (UBI) protein dissolved in aqueous solution. The Article is organized as follows: In the next section, we briefly recall the DFTB approach, and we formulate the coupling between the DFTB and FQ portions for both ground-state and excitation energies calculations. DFTB/FQ is then applied to the calculation of the excitation energies of DOX, the DOX–DNA complex, and the UBI protein in aqueous solution. Conclusions and perspectives end the Article.

## 2 Theory

In this section, the theoretical background of the DFTB/FQ approach is described. To this end, the fundamentals of DFTB and polarizable FQ approaches are briefly recapped, and the formulation of the DFTB/FQ coupling is presented. Then, the extension of the model to the linear response in a TD framework is discussed.

### 2.1 DFT Basis of TB Theory

In the general DFT framework, the energy functional in the KS picture reads^49^
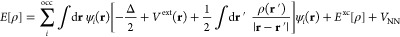
1where ψ_*i*_ are occupied KS eigenstates, Δ is the Laplacian operator, *V*^ext^ is the external potential associated with the nuclei–electron interaction, *E*^xc^ is the exchange-correlation contribution, and *V*_NN_ is the nuclei–nuclei repulsion term.

In the DFTB theory, the electronic density ρ is expressed as ρ = ρ_0_ + *δρ*, where ρ_0_ is a reference input density and *δρ* is a fluctuation, which is assumed to be small.^16,19,50^ Within this assumption, the exchange-correlation energy contribution can be expanded in a Taylor expansion, and eq 1 becomes
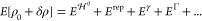
2
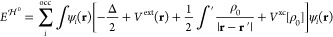
3
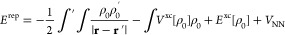
4
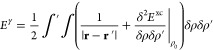
5
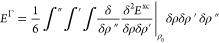
6where we have introduced the expectation value of the zeroth-order Hamiltonian  (which depends only on the reference density ρ_0_) and the so-called repulsive energy contribution *E*^rep^. *E*^γ^ and *E*^Γ^ collect the second- and third-order energy terms. Notice that in eqs 3–6, the usual shorthand notation such that , *δρ* = *δρ*(**r**), , *δρ*′ = *δρ*(**r**′), and so on, is used.^16,19,50^

Different DFTB methods can be defined by truncating the Taylor expansion in eq 2 at different orders. The most basic approach consists of neglecting *E*^γ^ and *E*^Γ^ in eq 2. This gives rise to a set of non-self-consistent KS equations because the zeroth-order Hamiltonian depends only on the reference density, ρ_0_. The repulsive energy *E*^rep^ is approximated as a sum of repulsive, short-ranged, two-body potentials, defined in terms of a set of parameters.^51^ The Hamiltonian  and overlap *S*_*μν*_ = ⟨ϕ_μ_|ϕ_ν_⟩ matrix elements are calculated at a set of relevant interatomic distances and are tabulated. By this, they do not need to be computed for each DFTB calculation, and this results in substantial computational savings as compared with standard DFT. Notice that various parametrizations for *E*^rep^ and the  and *S*_*μν*_ matrix elements have been proposed.^52^

A more sophisticated DFTB method, the SCC-DFTB, can be obtained by retaining *E*^γ^ in eq 2. The density fluctuation *δρ* is expressed as a sum of localized atomic contributions, *δρ* = *∑*_α_*δρ*_α_, which are subsequently approximated through the monopolar term of a multipolar expansion,^18^ that is,
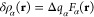
7where *F*_α_(**r**) is a normalized spherical density fluctuation centered on the αth atom, whereas the net charge *Δq*_α_ = *q*_α_ – *q*_α_^0^ is computed through a Mulliken charge analysis. Within such an assumption, *E*^γ^ can be rewritten as
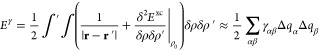
8where γ reads
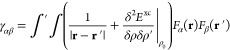
9Therefore, the total Hamiltonian matrix  can be written as
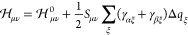
10Δ*q*_ξ_ explicitly depends on MO coefficients through the density matrix, thus introducing a nonlinearity in the Hamiltonian. As a result, DFTB equations must be solved iteratively.

Last, the third-order term *E*^Γ^ in eq 2 may also be retained, such as in the DFTB3 approach.^53,54^

### 2.2 DFTB/FQ Approach

As stated in the Introduction, in this work, DFTB is coupled to the polarizable FQ force field, which represent each classical atom in terms of a charge *q*, which is allowed to “fluctuate” so as to fulfill the electronegativity equalization principle, which states that the instantaneous electronegativity χ of each atom must be the same at equilibrium. The total charge on each FQ molecule is fixed to a certain value *Q* by using Lagrangian multipliers λ. The FQ energy can be written as^47^
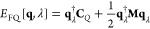
11where **q**_λ_ is the vector of FQ charges and Lagrange multipliers, **C**_*Q*_ is a vector collecting atomic electronegativities and charge constraints *Q*, and the **M** matrix takes into account the interaction kernel between FQ charges and Lagrangian blocks. In particular, the diagonal elements of the FQ–FQ block of **M** account for the charge self-interaction by means of the chemical hardness η.^45^ The minimization of the energy functional in eq 11 leads to a set of linear equations; their solution yields the FQ charges, that is
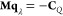
12Within a two-layer QM/MM scheme, the total energy of the DFTB/FQ system is written as
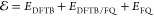
13where *E*_DFTB_ and *E*_FQ_ represent the energies of the DFTB and FQ portions and *E*_DFTB/FQ_ is the interaction energy between the two layers. Here, similarly to most QM/MM approaches, a purely classical interaction term is considered; that is, the DFTB and FQ portions interact through the electrostatic potential generated on the FQ charges by the *total* DFTB density, that is, the reference density ρ_0_ and the density fluctuation *δρ*. Within the DFTB framework, the QM/FQ interaction can be approximated by only taking into account the potential generated by *δρ*, similar to alternative DFTB/classical couplings.^55−57^ Therefore, the corresponding approximated molecular electrostatic potential at the *i*th FQ charge placed at **r**_*i*_ can be written as
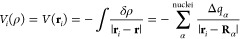
14where the implicit dependence of the electric potential on the density matrix through Mulliken charges is highlighted. Notice that in eq 14, the integration over the normalized spherical density fluctuations *F*_α_(**r**) should be included. (See eq 7.) However, because the distance between FQ charges and QM atoms is typically larger than any intramolecular distance, we can safely assume that *F*_α_(**r**) = δ(**r** – **R**_α_). Therefore, density fluctuations can be described through a set of Mulliken point charges.

Moving back to the total DFTB/FQ energy functional, it can be rewritten as
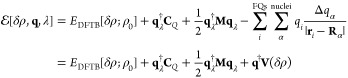
15Minimization of the energy functional in eq 15 with respect to both charges and Lagrangian multipliers yields the following linear system
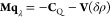
16The right-hand side of eq 16 collects both atomic electronegativities and the electric potential generated by the DFTB density. The latter term accounts for the mutual polarization among the DFTB and FQ portions of the system. In fact, KS equations need to be modified to include the DFTB/FQ contribution to the Hamiltonian matrix, which reads
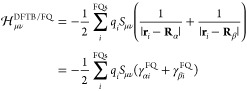
17where the kernel γ^FQ^ that takes into account the interaction between the αth Mulliken charge (or the basis function μ) and the *i*th FQ charge. Notice that the DFTB/FQ term to the total energy and Hamiltonian matrix is the same for both the SCC-DFTB and DFTB3 methods because the Mulliken-based expansion for the density fluctuation *δρ* does not change. Note finally that the formulation presented above is not limited to FQ but can easily be extended to any kind of variational polarizable MM approach.^58^

### 2.3 Linear Response Regime

The extension of the approach to the linear response regime allows the calculation of some spectral signals and, in particular, vertical transition energies and absorption spectra. The TD-DFTB eigenproblem can be expressed in the Casida formalism as^18^
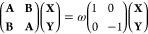
18where the eigenvalues ω correspond to excitation energies and the eigenvectors **X** and **Y** correspond to single-particle excitations and de-excitation amplitudes. Similarly to DFT/FQ,^34,59−63^ to take into account the FQ layer, we need to modify the DFTB response matrices **A** and **B** as follows
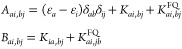
19where the indices *i*, *j* and *a*, *b* run over the occupied and virtual molecular orbitals with energies ε. *K*_*ia*,*jb*_ and *K*_*ia*,*jb*_^FQ^ are the DFTB and FQ coupling matrices, respectively. *K*_*ia*,*jb*_ is usually simplified by exploiting the so-called γ-approximation,^18^ similarly to the ground state in SCC-DFTB. (See eq 7.) In such an approximation, the transition density *p*_*ia*_(**r**) = ψ_*i*_(**r**)ψ_*a*_(**r**) is decomposed as a sum of atomic contributions that, after a multipolar expansion, is approximated by means of the monopole term only. (See also eq 7.) Therefore, *K*_*ia*,*jb*_ reads
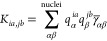
20where *q*_α_^*ia*^ and *q*_β_^*jb*^ are Mulliken atomic transition charges. The γ̅ functional is defined as in eq 9; however, the functional derivative of *E*_xc_ is evaluated on ρ. For systems with small charge-transfer effects, γ slightly depends on atomic charges, so that γ̅_*αβ*_ can be approximated with its ground-state counterpart γ_*αβ*_.

By following refs (34), (45), and (47), the FQ contribution to the coupling matrix can be defined as
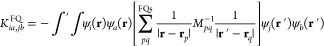
21At this point, we can exploit the DFTB γ approximation, and eq 21 becomes
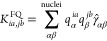
22where γ̂_*αβ*_ is defined as
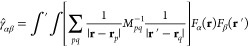
23Similar to the ground state, we can assume *F*_α_(**r**) = δ(**r** – **R**_α_). Thus we obtain
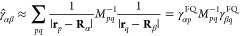
24where the interaction kernel defined in eq 17 is considered.

## 3 Computational Details

The equations presented in the previous section were implemented in a modified version of the Amsterdam Molecular Suite (AMS), release 2020.202 program.^23,64^ TD-DFTB/FQ calculations were performed on 200 configurations extracted from MD simulations already reported in the literature.^65−67^

For DOX in the gas phase, we ran calculations on the same conformations coming from the MD after removing surrounding water molecules. Table 1 lists an inventory of the different systems/environments addressed in this Article and some other results that will be presented in the following discussion.

**Table 1:** Inventory of the Number of Atoms, Water Molecules Described at the FQ Level, and Absorption Band Maxima (in Electronvolts) of All Systems under Study^a^

system	*N*_atoms_	*N*_FQwaters_	Abs_SCC_	Abs_DFTB3_
DOX in gas phase	69	0	2.41	2.43
DOX in water	3714	1215	2.33	2.35
DOX/water/DNA	9756	3100	2.37	2.36
UBI in gas phase^b^	1231	0	5.31, 4.75	5.37, 5.05, 4.77
UBI in water	11 731	3500	5.33, 4.80	5.43, 4.86

a Experimental results for the π → π* transitions of DOX and UBI in aqueous solution are 2.58 and 4.51 eV, respectively.^68,69^.

b From the geometry reported in ref (23).

To explore diverse DFTB Hamiltonians, we relied on the Slater–Koster-based DFTB class and performed TD-DFTB calculations for the entire set of snapshots using both the second-order self-consistent charge extension SCC-DFTB (recently also called DFTB2) and the third-order extension known as DFTB3. These two DFTB schemes are thoroughly explained elsewhere (see, e.g., ref (70)), but in short, SCC takes into account density fluctuations with improvements in the description of the polar bonds; likewise, DFTB3 describes hydrogen-bonded complexes and proton affinities, although at a little higher computational cost than SCC-DFTB calculations. SCC-DFTB and DFTB3 TD-DFTB calculations were performed by using mio-1-1^71^ and 3ob-3-1^72^ parameter sets, respectively.

Finally, to reduce the computational effort of TD-DFTB/FQ-based absorption spectra calculations, we tested the oscillator-strength-based truncation of the single-orbital transition space following the procedures introduced in a previous study.^23^ A summary of the technical settings used in the TD-DFTB calculations can be found in Table S1 in the Supporting Information (SI). In all of the cases, absorption spectra profiles were obtained through a convolution of the TD-DFTB excitations by using Gaussian line shapes with a full width at half-maximum (fwhm) value of 0.3 eV, if not explicitly stated. A minimum number of 100 excited states were converged in each calculation. In all calculations, we exploited the FQ parameters proposed in ref (73).

## 4 Numerical Results

In this section, we apply DFTB/FQ to describe absorption spectra. We analyze the effect that different choices of the DFTB Hamiltonian and the radius of the DFTB shell have on the spectra and test the accuracy of various intensity selection thresholds for the single orbital transition basis. Also, TD-DFTB/FQ spectra are compared with those obtained by using TD-DFTB calculations in the gas phase.

**Figure 1:**
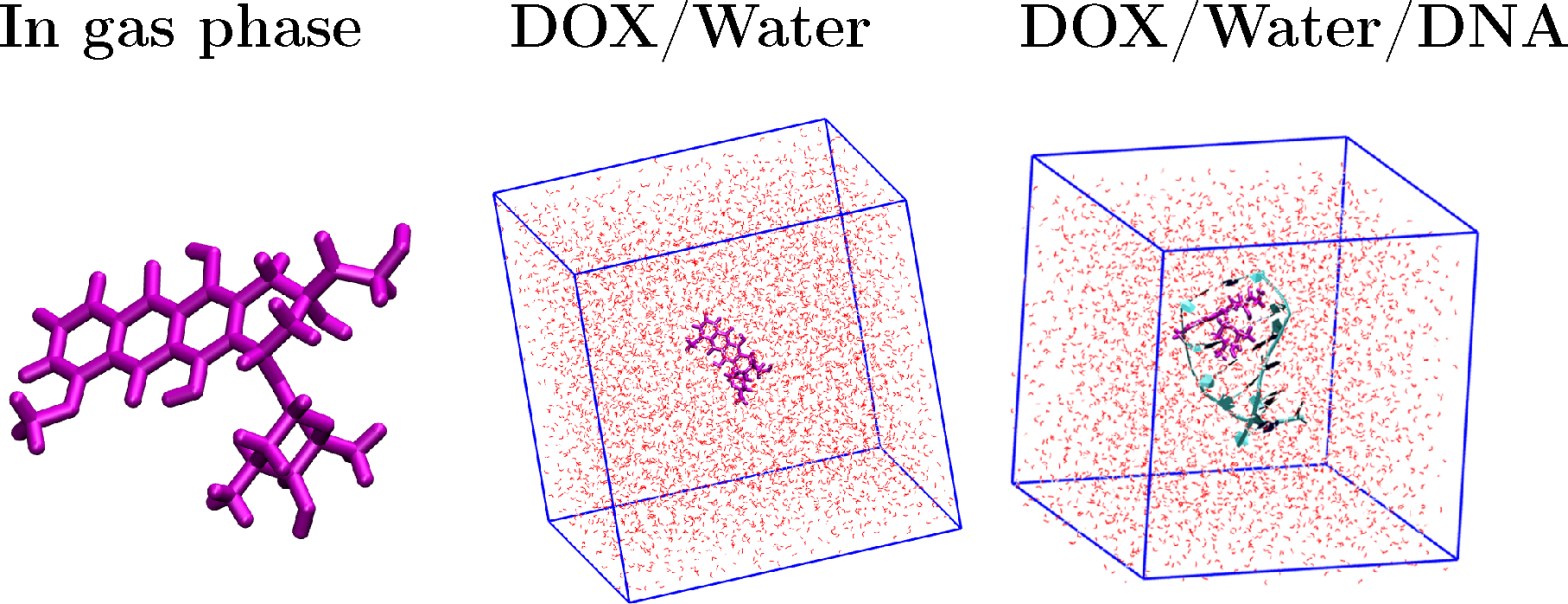
Three environments in which UV–vis spectra of doxorubicin were computed in this work. Left: Gas phase. Middle: Snapshot of the molecular dynamics of solvated DOX. Right: Snapshot of the molecular dynamics of DOX intercalated into DNA and surrounded by water molecules.

**Figure 2:**
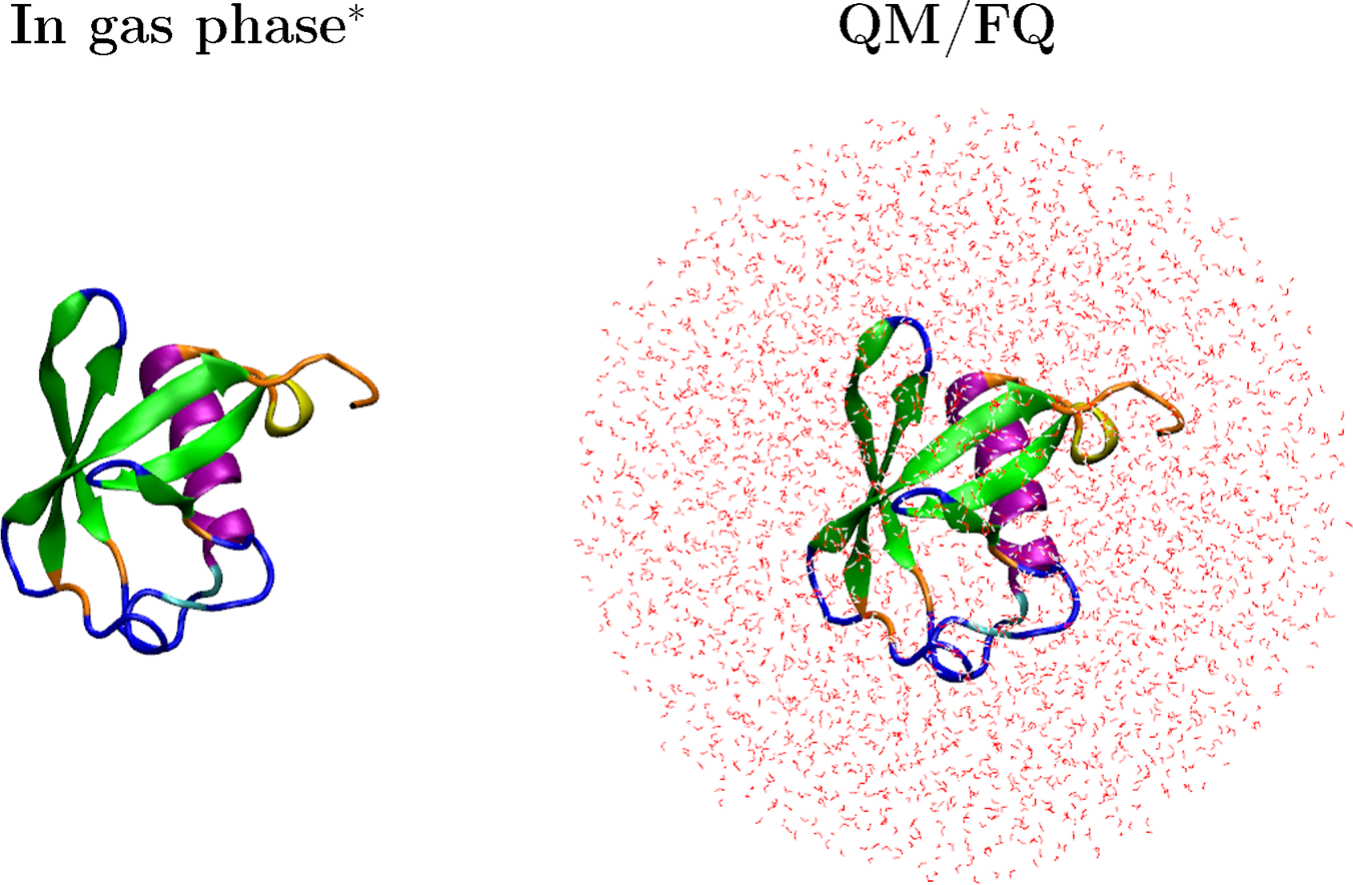
Environments in which the UV–vis spectra of ubiquitin were computed in this work. Left: Gas-phase conformation by using the same geometry reported in ref (23). Right: Snapshot of the molecular dynamics of solvated UBI is shown, which is treated with the QM/FQ approach.

As test cases, we have chosen two biologically relevant, flexible organic molecules, namely, DOX and UBI, whose structures are depicted in the left panels of Figures 1 and 2, respectively.

### 4.1 Doxorubicin

DOX is an anticancer drug,^74^ and it is commonly studied in the context of intercalation into DNA due to the proposed mechanism of action based on the insertion of its planar aromatic chromophore portion between sequential base pairs (BPs).^75−77^ The drug in water is quite investigated as well given that it travels from the purely aqueous environment to penetrate DNA helices.^65,68,78−81^ Regarding the DOX/DNA/water tertiary system, binding energy studies have shown that DOX affinity is sequence-dependent.^82^ Although the preferential binding of DOX to double-stranded (ds) DNA is still a subject of debate, recent works reported that among some hexameric evaluated sequences, DOX prefers to bind to the d(CGATCG) in the case of the 1:1 complexes.^67^ Therefore, we only discuss the intercalation complex of DOX with that DNA model.

Because of its importance as a chemotherapy medication, there are plenty of works dealing with the spectroscopic evidence of the insertion of a DOX molecule between pairs of nitrogen-containing nucleobases and for the spectral signatures of DOX in aqueous solution. Thus theoretical^65,80,81,83^ and experimentally^84−88^ obtained absorption spectra can be found in the literature for both environments. The main absorption band around 480 nm has been attributed to a π → π* transition,^68,86^ and some bathochromic and hypochromic effects are reported to occur upon intercalation.^86,89^ Notwithstanding, it is difficult to observe those shifts because there is a vibronic component dominating the shape of the band.^65^ The three environments in which we studied the absorption spectra of DOX are displayed in Figure 1.

**Figure 3:**
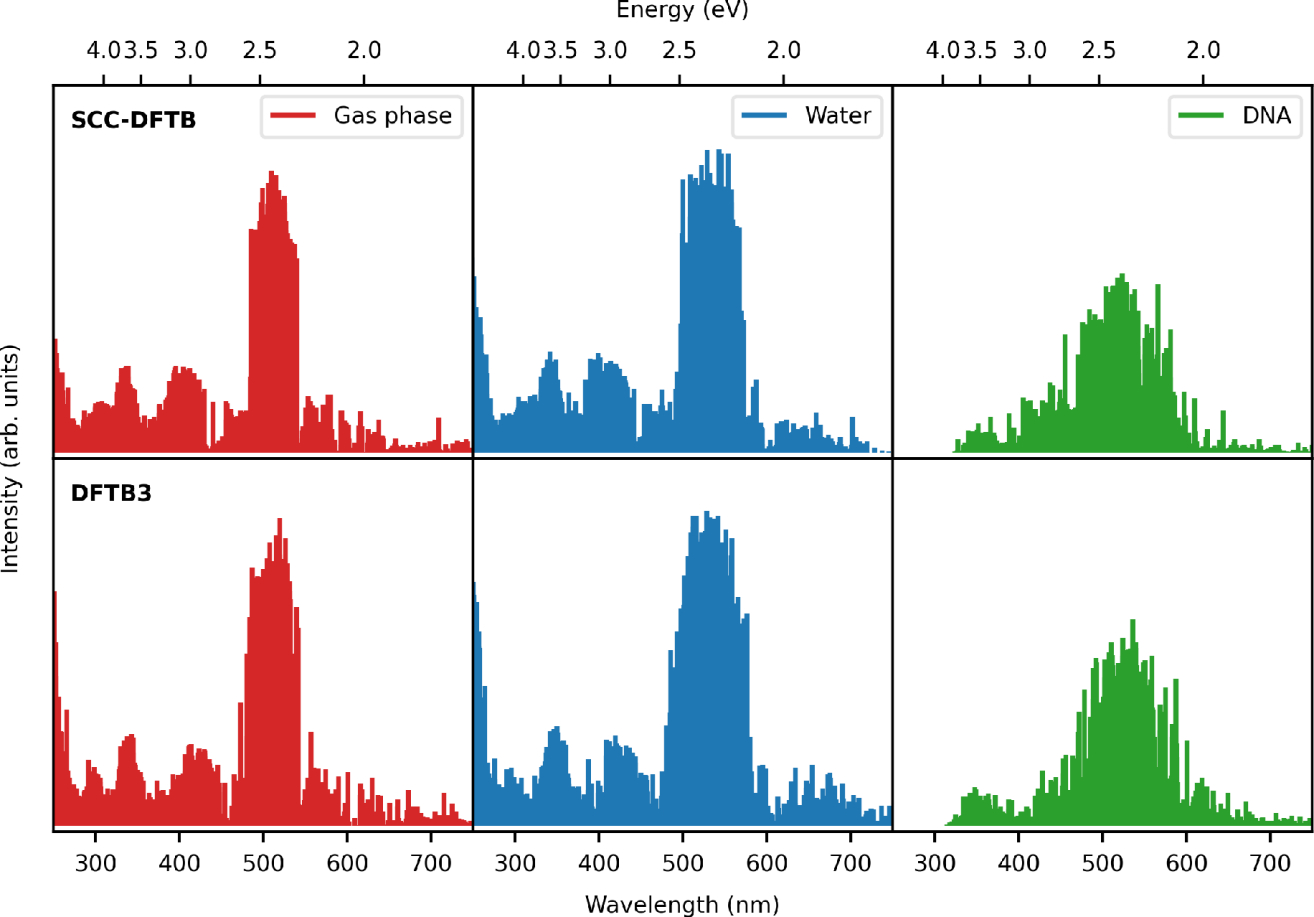
Stick spectra of doxorubicin *in vacuo* (red line), in water (blue line), and in DNA (green line) performed with different choices of the DFTB Hamiltonian.

**Figure 4:**
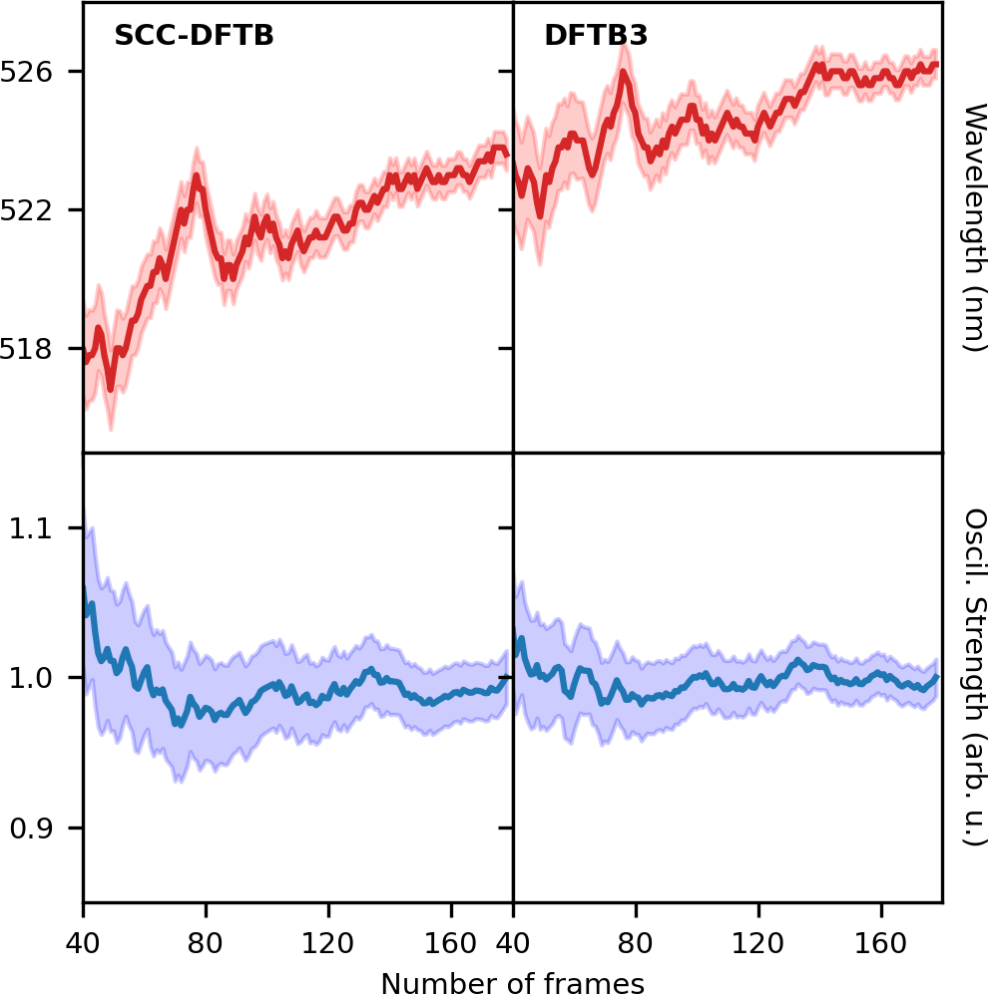
Convergence test for the absorption spectra of the tertiary DOX/water/DNA system. The position of the first excitation energy (top panel, red line) and the associated intensity (bottom panel, blue line) calculated with SCC-DFTB and DFTB3 model are reported. As a measure of the convergence, the 99% confidence intervals are reported.

From a set of snapshots (like those shown in Figure 1), the array of oscillator strengths obtained at their respective peak positions yields stick spectra (see Figure 3) with a natural broadening coming from the dynamical conformations of the chromophore and from the arrangements of the different molecules surrounding the system, that is, water molecules and the DNA basis. Computed stick spectra in the whole range of wavelengths are reported in Figure S1. It should be noted that the intensities of the sticks match the hypochromic effect reported to take place once the intercalation complex is formed. Furthermore, considering that the quality of the results depends on whether there is a convergence of the desired property, some test computations on the UV–vis spectra of DOX in the more complex environment were also performed with an increasing number of snapshots extracted from the MD. Figure 4 shows the convergence of the energy and intensity of the first electronic transition with respect to the number of frames along with the associated 99% confidence intervals. The convergence behavior of the total spectra with respect to the number of frames is reported in Figure S2.

In addition, we evaluated the effect that solute–solvent nonelectrostatic interactions (neglected in the pure DFTB/FQ method) might have on the absorption spectra by adding the solute’s closest water molecules to the QM portion and treating them with one of the DFTB Hamiltonians, whereas we described the remaining solvent molecules by means of FQ. This was done only for DOX in aqueous solution, and the results, together with the average number of water molecules (*N*_QM_) for each radius threshold (*R*), are reported in Table 2. Clearly, the role of nonelectrostatic (mainly repulsion) effects is minimal.

**Table 2:** Dependence of the Maximum Absorption Energies of Solvated DOX on the Size of the QM Shell^a^

*R* (Å)	*N*_QM_	ΔVEE_SCC_ (eV)	ΔVEE_DFTB3_ (eV)
1	0	0.00	0.00
2	10	0.01	0.01
3	47	0.01	0.01
4	85	0.02	0.01
5	126	0.02	0.01
6	180	0.03	0.02

a ΔVEE is the energy difference with the maximum absorption calculated at QM/FQ level. *N*_QM_ is the average number of water molecules treated in the QM portion.

#### 4.1.1 DFTB Model Hamiltonians

**Figure 5:**
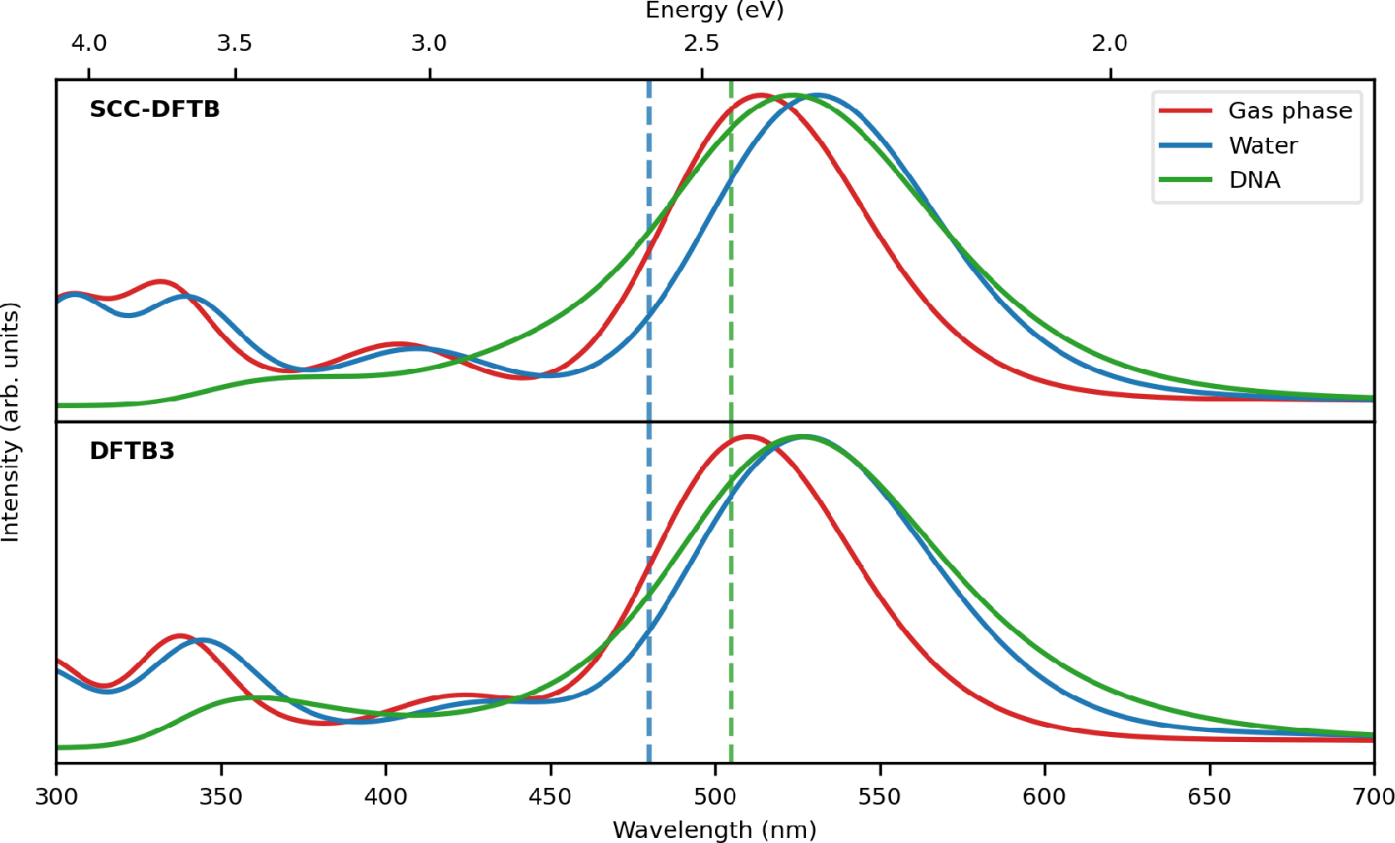
Absorption spectra of doxorubicin *in vacuo* (red line), in water (blue line), and in a water/DNA mix (green line) performed with different choices of the DFTB Hamiltonian. Experimental excitation energies from refs (68) and (86) in water and in water/DNA mix are reported with dashed lines.

**Figure 6:**
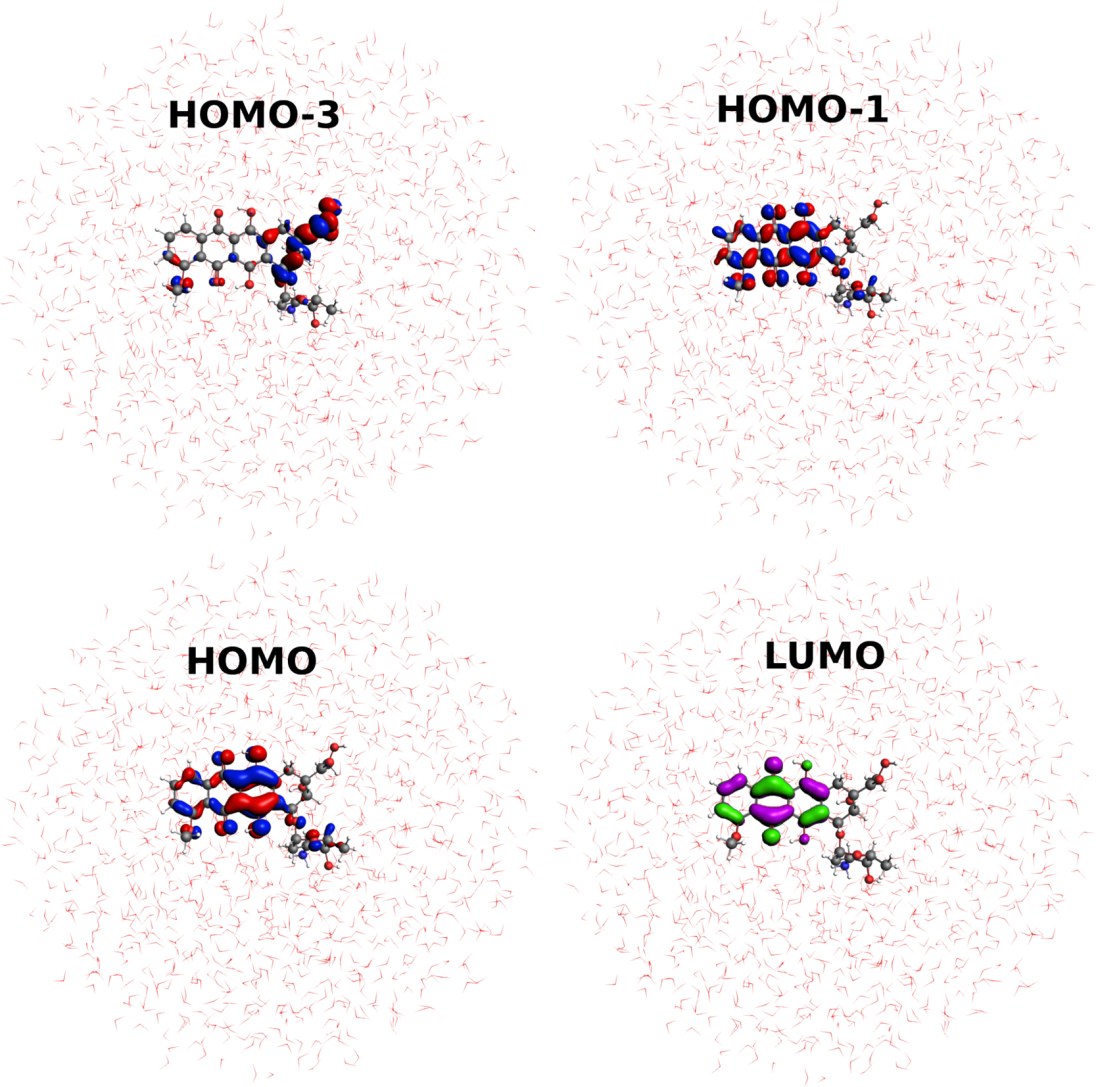
Most relevant MOs involved in the solvated doxorubicin absorption spectrum. For visualization purposes, virtual orbitals are depicted in different colors.

As mentioned previously, we exploited two of the classic Slater–Koster-based DFTB Hamiltonians, SCC-DFTB and DFTB3. The resulting DFTB/FQ normalized absorption spectra obtained from the average of ∼180 structures of DOX in different environments are plotted in Figure 5. Table 1 also contains the maximum absorption energies, as obtained from the averaged spectra for both DFTB schemes and for all of the DOX environments under study. Interestingly, both DFTB Hamiltonians offer a similar description of the absorption spectra and the main band attributed to the π → π* transition of the anthracycline chromophore. It should be emphasized that regardless of the DFTB model Hamiltonian and regardless of the environment, the HOMO and LUMO are, for the most part, the orbitals involved in the lowest energy transition. They are graphically depicted in Figure 6 along with other molecular orbitals belonging mainly to the rings of the DOX structure. These results are in line with those obtained by Olszówka et al.,^65^ who reported a single HOMO → LUMO transition to be responsible for the appearance of the main band in the absorption spectra.

As can be seen in Figure 5, the main environment effect is the red shift of the main band moving from the gas phase to water and water/DNA solutions. Overall, TD-DFTB/FQ reproduces the general shape of other published electronic absorption spectra,^81^ although the main band is red-shifted by ∼0.2 eV when compared with the experimental maximum absorption energy of solvated DOX. Discrepancies between calculated and experimental results have already been reported for such systems and moderately corrected by using the vertical gradient (VG) or adiabatic Hessian (AH) approaches to vibronically resolve the spectra.^65^ Also, as is reported in a recent paper,^90^ it would be beneficial to consider different DOX tautomers to obtain a full description of the absorption spectra.

**Figure 7:**
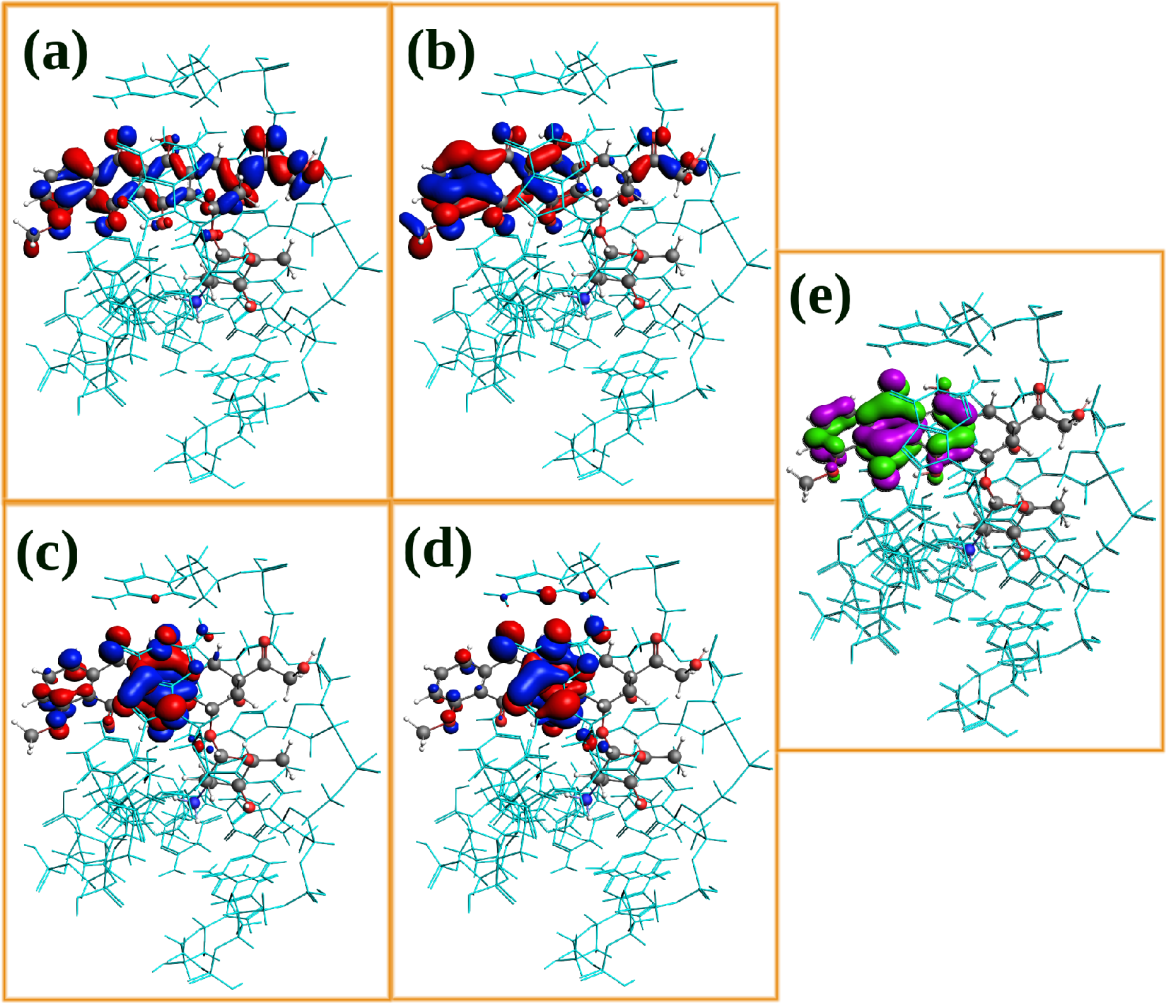
Most relevant MOs involved in the doxorubicin absorption spectrum when intercalated into DNA (represented by cyan sticks). (a–d) Occupied orbitals. (e) LUMO orbital. For visualization purposes, virtual orbitals are depicted in different colors.

Going from water to water/DNA, there are just slight differences in the vertical excitation energies with both Hamiltonians; however, the spectral profile does exhibit some changes, including a broader main band when DOX is intercalated into DNA and also a different spectral shape at higher energies where the nucleotides are also involved in the electronic transitions. To understand the root causes of these differences in the spectra, especially in the main peak, we have plotted in Figure 7 the molecular orbitals that play a pivotal role in that particular excitation. By analysis and comparison of these orbitals with those displayed in Figure 6, it becomes clear that the aromatic rings of the DOX’s nearest nucleotides are also participating in the transitions, although the majority of them include the anthraquinone rings (the portion that intercalates between two BPs of dsDNA) and the anchor domains of DOX, where the latter are responsible for stabilizing the DOX–DNA complex via hydrogen bonds with DNA bases. It is worth noting at this point that the HOMO of the ternary DOX/water/DNA system is not localized in the DOX molecule, unlike the situation in which DOX is in the aqueous solution environment.

#### 4.1.2 Vertical Excitation Energy Dependence on the Size of the DFTB Shell

**Figure 8:**
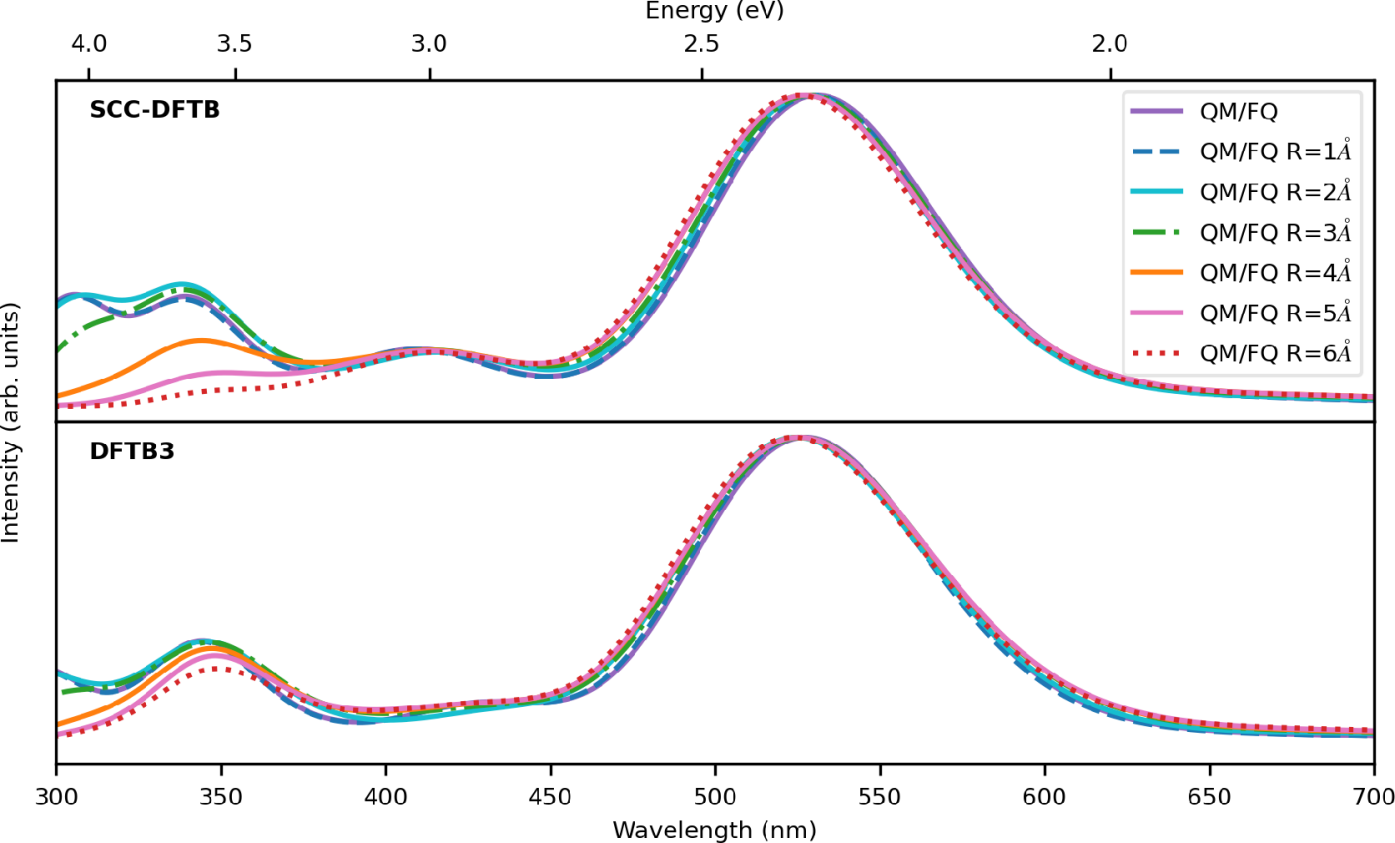
TD-DFTB/FQ absorption spectra of doxorubicin in water, varying the size of the QM portion and using two different DFTB Hamiltonians. The radius of the DFTB shell is reported in the key.

As shown in Figure 8, spectra obtained by varying the number of water molecules in the DFTB layer (QM/QM*w*/FQ) do not substantially differ each other, which is also confirmed by the data reported in Table 2, where ΔVEE, that is, the difference in vertical excitation energy (VEE), does not exceed 0.03 eV when compared with the QM/FQ result. It can therefore be argued that regardless of the Hamiltonian choice, the inclusion of the solvent does not play a pivotal role in the description of the bright π → π* transition of solvated DOX; however, the spectral profiles look dissimilar at shorter wavelengths, with a more pronounced contrast in the SCC case.

#### 4.1.3 Intensity Selection Thresholds

**Figure 9:**
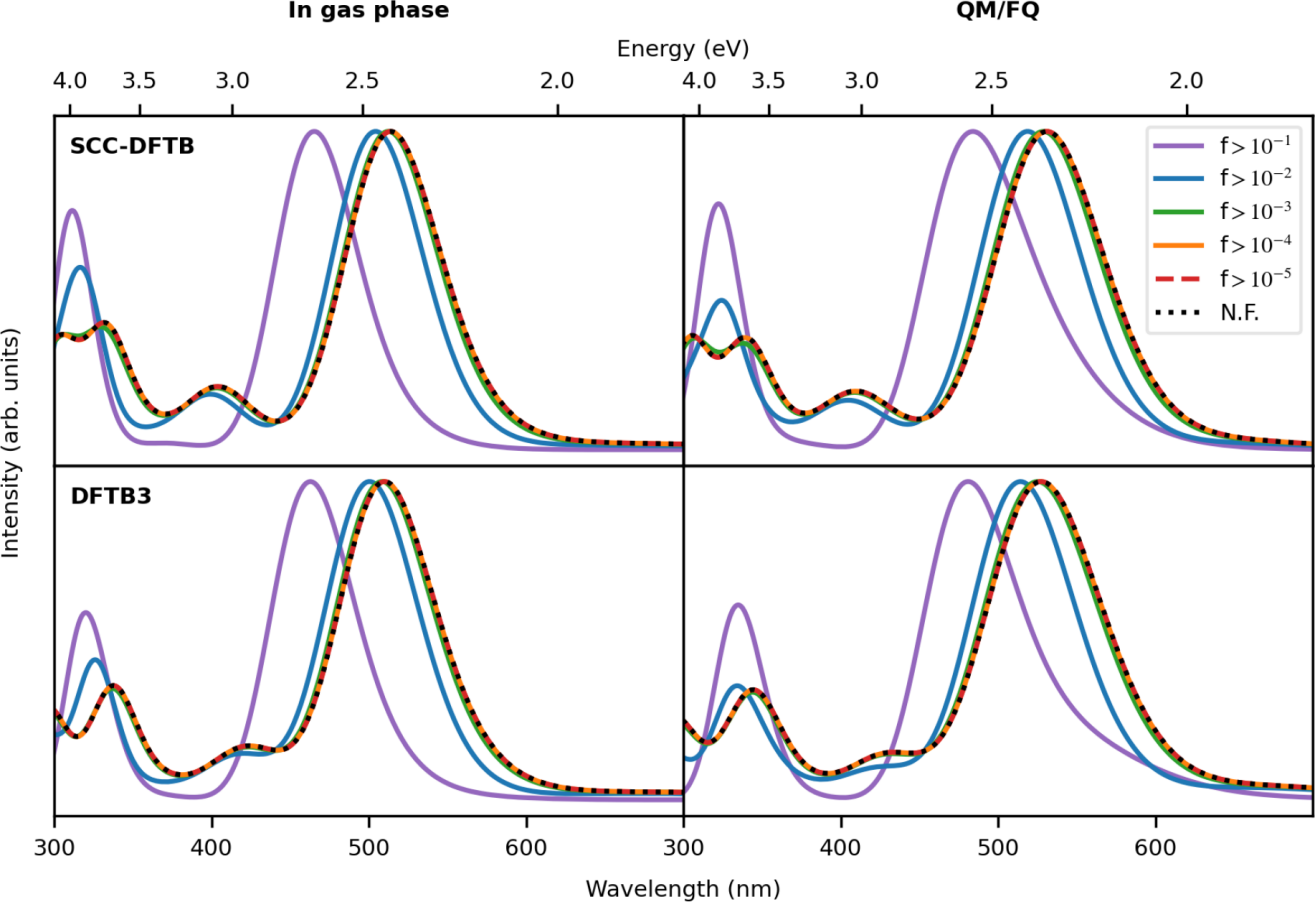
TD-DFTB and TD-DFTB/FQ absorption spectra of doxorubicin *in vacuo* (left panel) and in water (right panel) performed with different choices of the DFTB Hamiltonian and changing the intensity-selection thresholds for the single-orbital transitions basis. N.F. stands for no filter or that all single orbital transitions were considered.

Figure 9 shows TD-DFTB calculated absorption spectra of DOX in the gas phase and in aqueous solution, obtained with intensity selection at different oscillator strength thresholds. It should be noticed that the reduced computational cost of the intensity-selected TD-DFTB leads to a loss in accuracy because there is a blue shift of the main band for larger thresholds. (See, for instance, *f* > 0.1 and *f* > 0.01.) Nevertheless, when a filter smaller than 0.001 is used, it is evident that the truncation of the basis in oscillator strength has a relatively small effect on the absorption spectrum. In fact, the relative intensities, number of peaks, and peak positions are kept, and the spectrum is practically unaltered compared with the nonfilter case, which is valid for both Hamiltonians. These findings indicate that a large part of the basis has a minor contribution to the spectra, as already reported for the simulation of the absorption spectra of C_60_ fullerene, Ir(ppy)_3_, and UBI.^23^

### 4.2 Ubiquitin

UBI is a 76-amino acid polypeptide (1231 atoms) with diverse roles, mainly oriented to help in the regulation of the processes of other proteins in the body.^91−94^ This small protein has been considered as a universal constituent of living cells.^95^ Structurally speaking, UBI contains important chromophores like tyrosine and phenylalanine, with the former presenting higher absorbance. As a matter of fact, UBI has served as a model protein to study the sensitivity of UV–visible spectroscopy to environmental factors.^69,96^

DFTB/FQ is challenged in this section to compute the UV–vis absorption spectra of UBI in aqueous solution. The entire protein has been treated at the DFTB level, whereas water molecules are described by means of the FQ force field. Two major features are visible in the UBI experimental spectra in solution and in the gas phase, as reported by Bellina et al.:^69^ (i) a broad band centered around 275–280 nm and (ii) an intense response at high energy with an onset at 250 nm. Indeed, it has been found that aromatic amino acids and proteins absorb UV light and show two main bands in UV–vis spectra, one centered on 280 nm that is the result of absorbance by the aromatic ring portion of their structure and a second one at lower wavelengths, which stems from the absorbance of peptide and carboxylic acid moieties. Because of this, it is not surprising that for UBI, a polypeptide containing tyrosine, the same bands are found. In particular, when Tyr is in aqueous solution, absorption maxima appear at ∼220 (higher absorbance) and 275 nm;^97−99^ some authors have postulated that the two bands are probably arising from two well-separated π → π* transitions.^100,101^

**Figure 10:**
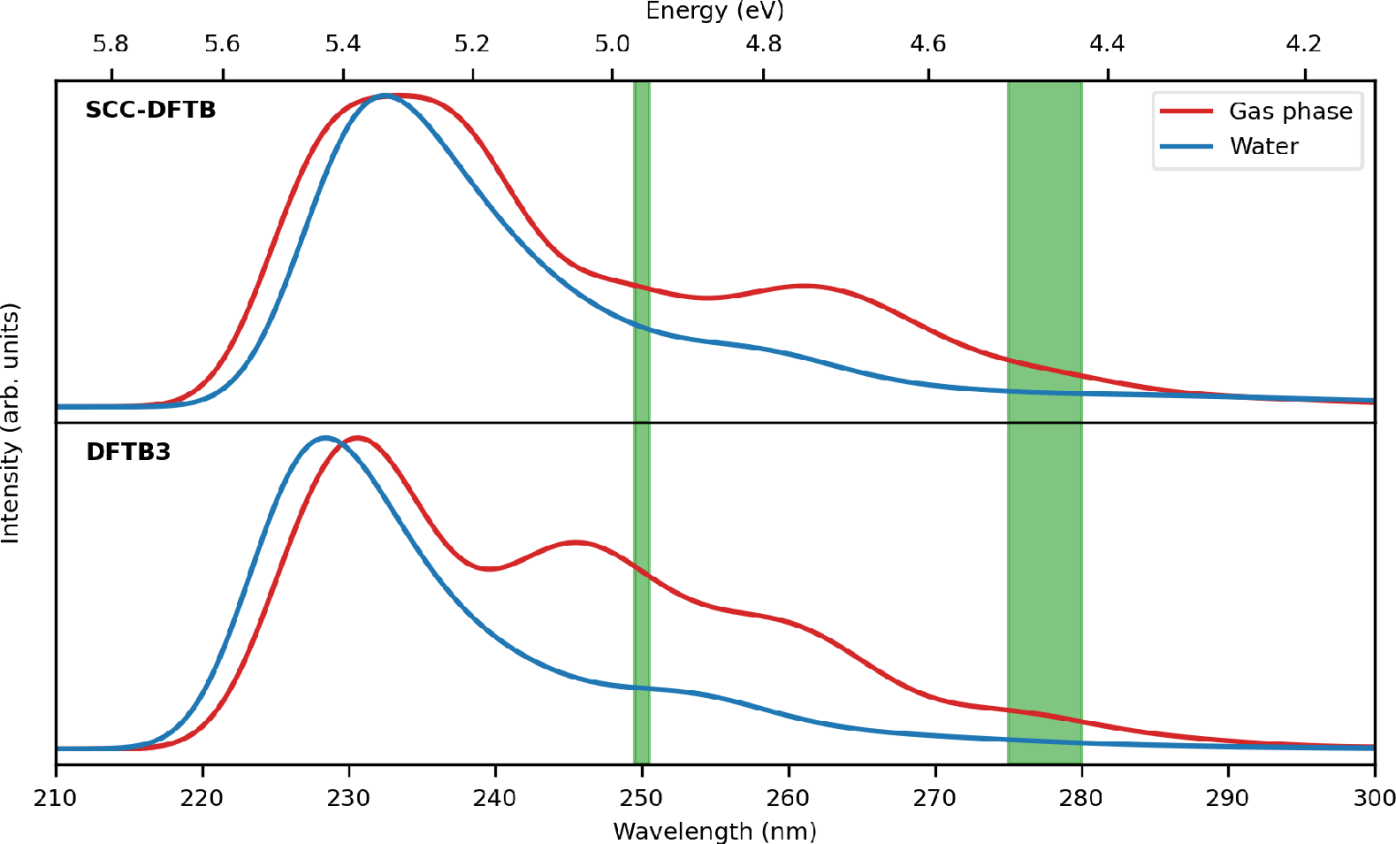
Comparison between calculated absorption spectra of UBI in the gas phase and aqueous solution, as obtained with different DFTB model Hamiltonians. The experimental data of UBI in the gas phase from ref (69) are reported with green blocks representing the experimental range of the two main absorption bands.

The influence of the environment on the absorption spectra of the UBI protein has already been demonstrated.^96^ This effect can also be observed in Figure 10, which shows the spectra in the gas phase and in aqueous solution and a comparison between the results coming from the two model Hamiltonians. Such spectra have been obtained through a Gaussian convolution of the TD-DFTB excitations using an fwhm value of 0.2 eV. As a reference, the computed stick spectra of UBI in the gas phase and in aqueous solution over the entire spectral range are reported in Figure S3.

**Figure 11:**
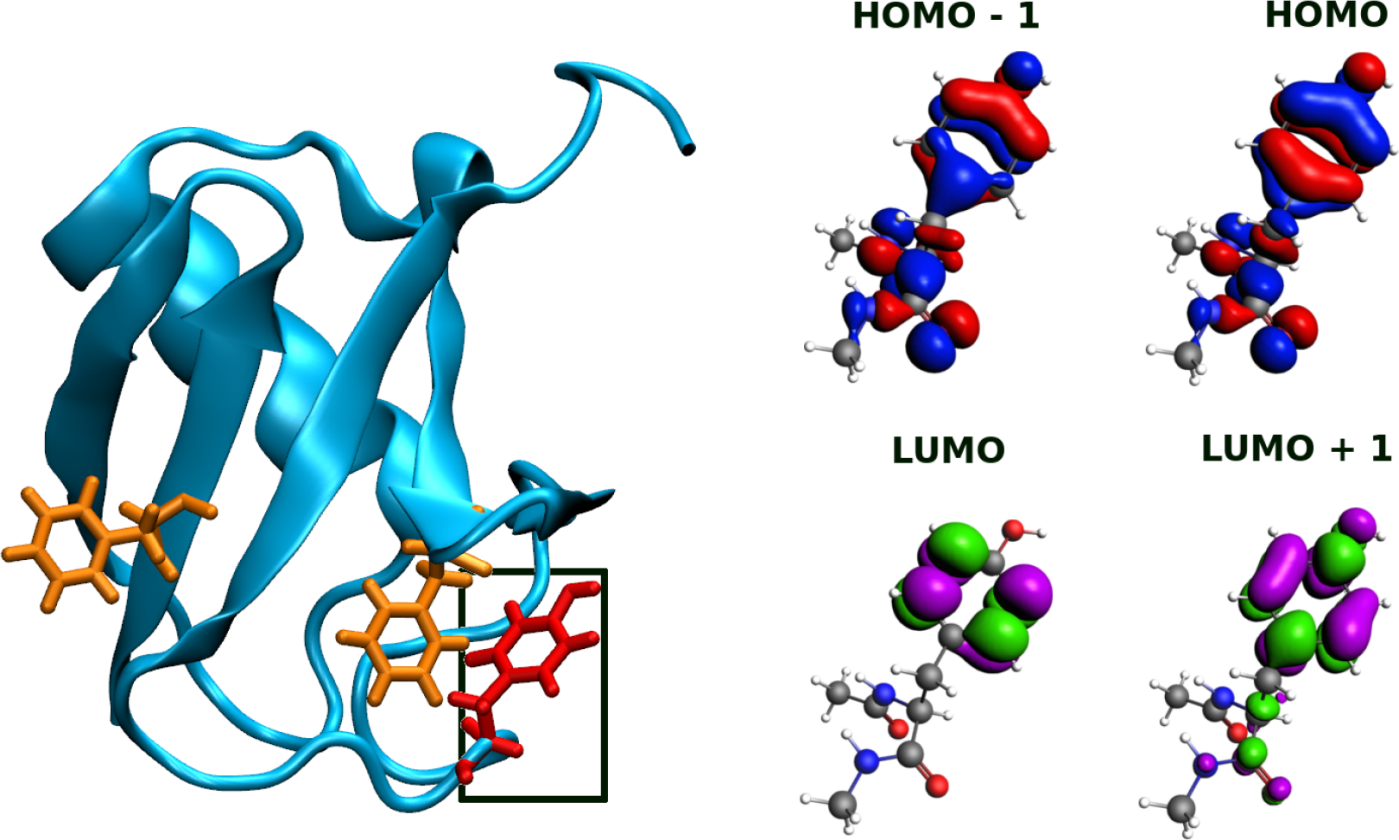
Left panel: Chromophores tyrosine (framed in black) and phenylalanine, which are the dominant sources of the UBI protein absorbance. Right panel: Molecular orbitals of extended Tyr mainly involved in the transitions. (See the text.) Extended Tyr means that in the calculations, the residue was capped with N-terminal acetyl and C-terminal N-Me amide capping groups to preserve the peptide bonds inside the protein. For visualization purposes, virtual orbitals are depicted in different colors.

It can be observed that both SCC and DFTB3 yield the same spectral shape in the case of solvated UBI, whereas a different behavior is observed in the gas phase. Such discrepancies may be attributed to different parametrizations exploited in the two approaches.^102^ Additionally, the presence of water has a tiny but non-negligible effect on the absorption spectra, overall red-shifting the main peaks that appear in gas phase spectra. The same calculations have also been performed by employing the nonpolarizable TIP3P force field to describe water molecules. (See Figure S4.) Absorption band maxima of UBI are also summarized in Table 1. To assign the transitions in the full protein, we applied a filter on the strongest oscillator strengths in the region of the maximum absorbance, and we quantified the contribution of single orbital transitions, thus identifying the leading contributing molecular orbitals and the part of the protein where they were situated. As has been indicated above, the band centered around 280 nm can be assigned to a π → π* excitation, and even though orbitals from very distinct residues can participate in the transitions, the predominant ones are localized on the aromatic tyrosine and phenylalanine chromophores, with tyrosine being responsible for most of the absorbance. A more in-depth analysis indicates that frontier molecular orbitals (HOMO, HOMO–1, LUMO, and LUMO+1) in the extended tyrosine residue resemble those involved in the absorption band at 280 nm of the UBI protein. The considered orbitals are presented in Figure 11 and are responsible for 90% of the state, having the greatest oscillator strengths in the region of the π → π* excitation.

We move on to compare our results with experimental data on spectral shifts going from solution to the gas phase. It was previously determined that the π → π* band in the gas phase is red-shifted as compared with the absorption in solution.^69^ In addition, it is known that tyrosine is located at the surface of the protein^103^ and thus has a strong effect on the environment, and the addition of water is also anticipated to cause a red shift of the tyrosine absorption spectrum.^104^ In this context, it should be noted that although our results are in agreement with these observations, the experimental final shift is not fully reliable due to the fact that UV gas-phase spectra were measured for the isolated deprotonated protein and UBI can change its conformation going from the gas phase to solution.^69^

**Figure 12:**
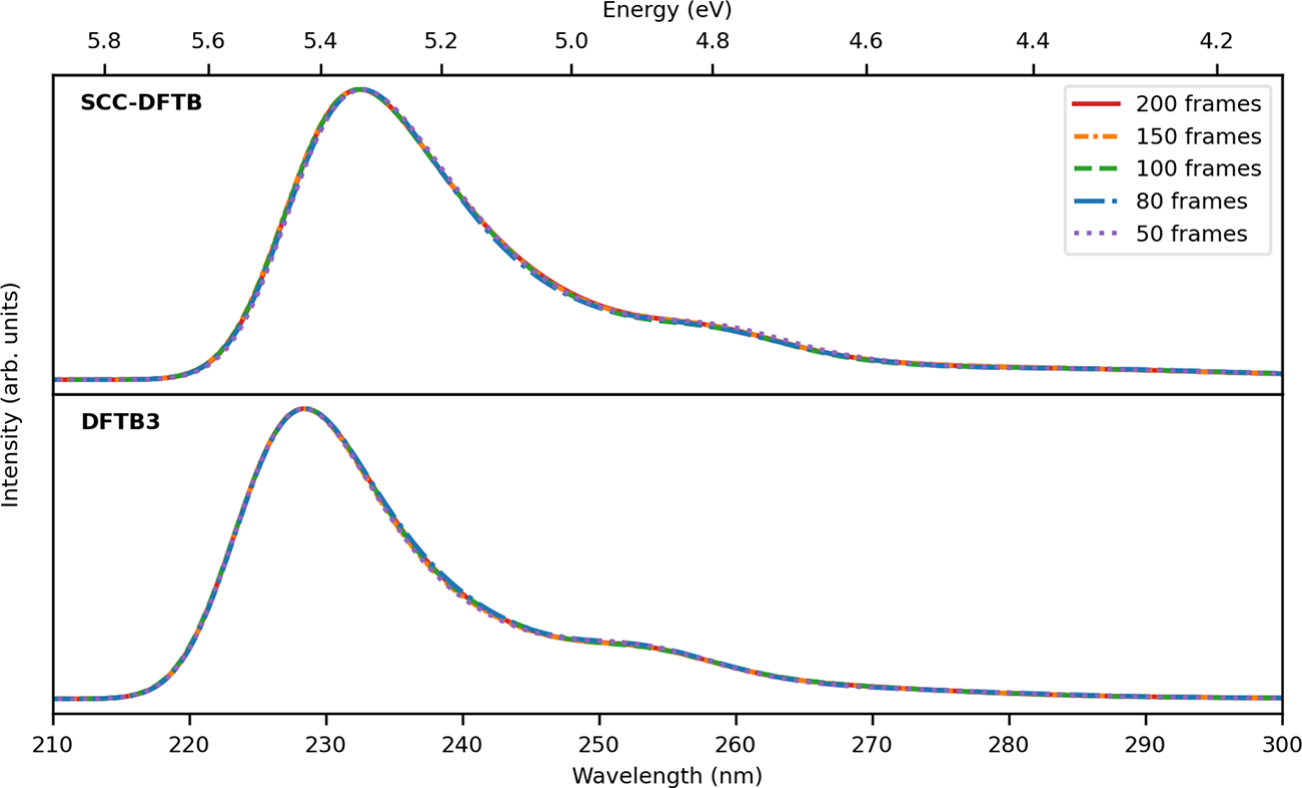
TD-DFTB/FQ absorption spectra of ubiquitin in aqueous solution, as obtained by averaging out an increasing number of snapshots.

Finally, we remark that it is necessary to ensure the analysis to be performed on final converged spectra. Inspection of UBI spectra in aqueous solution, as obtained by averaging out a varying number of frames (Figure 12), reveals that increasing the number of frames has no impact on the final band shape; in fact, a sampling of 50 frames is sufficient to achieve convergence, with no missing features appearing in the spectra. Lastly, if single orbital transitions with an oscillator strength smaller than 0.001 are removed (results not shown here), then the spectra do not change, and as expected, the computational effort decreases.

## 5 Summary, Conclusions, and Future Perspectives

We have presented a novel polarizable QM/MM approach where DFTB is coupled to the polarizable FQ force field. The model, which has been extended to TD-DFTB linear response, permits us to treat large systems in the condensed phase thanks to the favorable scaling of DFTB as compared with standard DFT and other *ab initio* methods. DFTB/FQ has been applied to the simulation of the electronic absorption spectra of DOX and UBI in different environments, showing that the inclusion of the FQ layer strongly affects spectral shapes and accounts for changes in both peaks’ positions and relative intensities when going from the gas phase to condensed phase.

The low computational cost of DFTB has allowed for a detailed analysis of the role of nonelectrostatic effects in the case of DOX in aqueous solution. In particular, the size of the DFTB portion has been varied by adding up a limited number of water molecules, showing that the DOX electronic response is almost unaffected by increasing the radius of the DFTB portion above 4 Å. This confirms the short-range nature of the nonelectrostatic interactions that are naturally included within the DFTB portion and that are dominated by Pauli repulsion. In addition, we show that by selecting the most intense spectral bands only, the accuracy of computed spectra is not particularly affected; however, the computational time of TD-DFTB/FQ calculations is substantially reduced. DOX and UBI spectral profiles have been obtained by taking into account solute and solvent dynamics; the phase-space sampling and the consequent configurational variability in both solute and solvent moieties have brought up natural broadening in absorption bands, which is directly obtained from the signals arising on the different snapshots extracted from MD simulations.

Finally, the results reported in this work show that the combination of DFTB and FQ permits us to model absorption spectra of large molecules embedded in complex environments at a low computational cost and in nice agreement with experimental data. This opens up the opportunity to explore more challenging spectroscopies in different environments. In fact, DFTB/FQ (similarly to other QM/polarizable MM approaches) can simulate molecular properties in any kind of complex environment, pending appropriate parametrization.

To validate the accuracy of DFTB/FQ to describe solvatochromic effects, we have also computed vertical excitation energies at the TD-DFT/FQ level (see Table S2), in line with the preliminary calculations of some of the present authors.^80,81,105^ DFTB/FQ and TD-DFT/FQ values are very similar, thus demonstrating that DFTB/FQ gives a correct description, from both a qualitative and quantitative point of view, for our systems. However, a more extended benchmark analysis on several systems would be required to finally validate the accuracy of the approach, as solvatochromic effects are strongly dependent on both the system and the nature of the excitation.

Improvement in the numerical performance of DFTB/FQ can be achieved by reparametrizing the FQ force field for DFTB calculations for both aqueous and nonaqueous solutions, in line with previous studies of some of the present authors.^46,106^ Also, the description of the environment can be refined by adding polarizable dipole moments on MM atoms, such as in the recently developed FQFμ approach, which appropriately simulated anisotropic solvent effects.^35,37^ In addition, in this Article, we have fully relied on a purely electrostatic model. Although electrostatics often dominates solvation effects, we have recently shown that nonelectrostatic interactions, in particular, Pauli repulsion, may strongly affect computed molecular properties.^107^ Such terms can be included in the DFTB/FQ approach by following recent works of our group.^48,107^

## Supporting Information Available

Technical settings for TD-DFTB/FQ calculations. Stick spectra of DOX *in vacuo*, in water, and in DNA/water computed at the TD-DFTB/FQ level. Convergence of the DOX/DNA/water absorption spectrum with the number of snapshots. Vertical excitation energies of DOX *in vacuo* and the DOX/water system computed at the TD-DFT/FQ and TD-DFTB/FQ levels. Stick spectra of UBI *in vacuo* and in aqueous solution. Absorption spectra of UBI/water computed at the TD-DFTB/TIP3P level (PDF)

## References

[ref1] Ratcliff L. E.; Mohr S.; Huhs G.; Deutsch T.; Masella M.; Genovese L. Challenges in large scale quantum mechanical calculations. WIREs Comput. Mol. Sci. 2017, 7, e1290 10.1002/wcms.1290.

[ref2] Dawson W.; Degomme A.; Stella M.; Nakajima T.; Ratcliff L. E.; Genovese L. Density functional theory calculations of large systems: Interplay between fragments, observables, and computational complexity. WIREs Comput. Mol. Sci. 2021, e1574 10.1002/wcms.1574.

[ref3] Ochsenfeld C.; Kussmann J.; Lambrecht D. S. Linear-scaling methods in quantum chemistry. Rev. Comput. Chem. 2007, 23, 1 10.1002/9780470116449.ch1.

[ref4] Ma Q.; Werner H.-J. Explicitly correlated local coupled-cluster methods using pair natural orbitals. WIREs Comput. Mol. Sci. 2018, 8, e1371 10.1002/wcms.1371. 29211961

[ref5] Li W.; Ni Z.; Li S. Cluster-in-molecule local correlation method for post-Hartree-Fock calculations of large systems. Mol. Phys. 2016, 114, 1447–1460. 10.1080/00268976.2016.1139755.

[ref6] Folkestad S. D.; Koch H. Multilevel CC2 and CCSD Methods with Correlated Natural Transition Orbitals. J. Chem. Theory Comput. 2020, 16, 179 10.1021/acs.jctc.9b00701. 31743013

[ref7] Marrazzini G.; Giovannini T.; Scavino M.; Egidi F.; Cappelli C.; Koch H. Multilevel density functional theory. J. Chem. Theory Comput. 2021, 17, 791–803. 10.1021/acs.jctc.0c00940. 33449681 PMC7880574

[ref8] Bennie S. J.; Curchod B. F.; Manby F. R.; Glowacki D. R. Pushing the limits of EOM-CCSD with projector-based embedding for excitation energies. J. Phys. Chem. Lett. 2017, 8, 5559–5565. 10.1021/acs.jpclett.7b02500. 29076727

[ref9] Manby F. R.; Stella M.; Goodpaster J. D.; Miller T. F. III A simple, exact density-functional-theory embedding scheme. J. Chem. Theory Comput. 2012, 8, 2564–2568. 10.1021/ct300544e. 22904692 PMC3419460

[ref10] Giovannini T.; Koch H. Energy-based molecular orbital localization in a specific spatial region. J. Chem. Theory Comput. 2021, 17, 139–150. 10.1021/acs.jctc.0c00737. 33337150

[ref11] Thiel W. Semiempirical quantum-chemical methods. WIREs Comput. Mol. Sci. 2014, 4, 145–157. 10.1002/wcms.1161.

[ref12] Thiel W. Theory and Applications of Computational Chemistry; Elsevier, 2005; pp 559–580.

[ref13] Dral P. O.; Wu X.; Thiel W. Semiempirical quantum-chemical methods with Orthogonalization and dispersion corrections. J. Chem. Theory Comput. 2019, 15, 1743–1760. 10.1021/acs.jctc.8b01265. 30735388 PMC6416713

[ref14] Bannwarth C.; Caldeweyher E.; Ehlert S.; Hansen A.; Pracht P.; Seibert J.; Spicher S.; Grimme S. Extended tight-binding quantum chemistry methods. WIREs Comput. Mol. Sci. 2021, 11, e1493 10.1002/wcms.1493.

[ref15] Seifert G.; Joswig J.-O. Density-functional tight binding—An approximate density-functional theory method. WIREs Comput. Mol. Sci. 2012, 2, 456–465. 10.1002/wcms.1094.

[ref16] Elstner M.; Porezag D.; Jungnickel G.; Elsner J.; Haugk M.; Frauenheim T.; Suhai S.; Seifert G. Self-consistent-charge density-functional tight-binding method for simulations of complex materials properties. Phys. Rev. B 1998, 58, 7260 10.1103/PhysRevB.58.7260.

[ref17] Gaus M.; Cui Q.; Elstner M. Density functional tight binding: application to organic and biological molecules. WIREs Comput. Mol. Sci. 2014, 4, 49–61. 10.1002/wcms.1156.

[ref18] Niehaus T. A.; Suhai S.; Della Sala F.; Lugli P.; Elstner M.; Seifert G.; Frauenheim T. Tight-binding approach to time-dependent density-functional response theory. Phys. Rev. B 2001, 63, 085108 10.1103/PhysRevB.63.085108.

[ref19] Elstner M. The SCC-DFTB method and its application to biological systems. Theor. Chem. Acc. 2006, 116, 316–325. 10.1007/s00214-005-0066-0.

[ref20] Jacquemin D.; Perpète E. A.; Scalmani G.; Frisch M. J.; Kobayashi R.; Adamo C. Assessment of the efficiency of long-range corrected functionals for some properties of large compounds. J. Chem. Phys. 2007, 126, 144105 10.1063/1.2715573. 17444699

[ref21] Jacquemin D.; Perpete E. A.; Scuseria G. E.; Ciofini I.; Adamo C. TD-DFT performance for the visible absorption spectra of organic dyes: conventional versus long-range hybrids. J. Chem. Theory Comput. 2008, 4, 123–135. 10.1021/ct700187z. 26619986

[ref22] Sokolov M.; Bold B. M.; Kranz J. J.; Höfener S.; Niehaus T. A.; Elstner M. Analytical Time-Dependent Long-Range Corrected Density Functional Tight Binding (TD-LC-DFTB) Gradients in DFTB+: Implementation and Benchmark for Excited-State Geometries and Transition Energies. J. Chem. Theory Comput. 2021, 17, 2266–2282. 10.1021/acs.jctc.1c00095. 33689344

[ref23] Rüger R.; van Lenthe E.; Lu Y.; Frenzel J.; Heine T.; Visscher L. Efficient Calculation of Electronic Absorption Spectra by Means of Intensity-Selected Time-Dependent Density Functional Tight Binding. J. Chem. Theory Comput. 2015, 11, 157–167. 10.1021/ct500838h. 26574214

[ref24] Paesani F. Getting the right answers for the right reasons: Toward predictive molecular simulations of water with many-body potential energy functions. Acc. Chem. Res. 2016, 49, 1844–1851. 10.1021/acs.accounts.6b00285. 27548325

[ref25] Reichardt C.; Welton T. Solvents and Solvent Effects in Organic Chemistry; John Wiley & Sons, 2010.

[ref26] Tomasi J.; Mennucci B.; Cammi R. Quantum mechanical continuum solvation models. Chem. Rev. 2005, 105, 2999–3094. 10.1021/cr9904009. 16092826

[ref27] Mennucci B. Polarizable continuum model. WIREs Comput. Mol. Sci. 2012, 2, 386–404. 10.1002/wcms.1086.

[ref28] Cappelli C. Integrated QM/polarizable MM/continuum approaches to model chiroptical properties of strongly interacting solute-solvent systems. Int. J. Quantum Chem. 2016, 116, 1532–1542. 10.1002/qua.25199.

[ref29] Curutchet C.; Muñoz-Losa A.; Monti S.; Kongsted J.; Scholes G. D.; Mennucci B. Electronic energy transfer in condensed phase studied by a polarizable QM/MM model. J. Chem. Theory Comput. 2009, 5, 1838–1848. 10.1021/ct9001366. 26610008

[ref30] Olsen J. M. H.; Kongsted J. Molecular properties through polarizable embedding. Adv. Quantum Chem. 2011, 61, 107–143. 10.1016/B978-0-12-386013-2.00003-6.

[ref31] Loco D.; Polack É.; Caprasecca S.; Lagardère L.; Lipparini F.; Piquemal J.-P.; Mennucci B. A QM/MM approach using the AMOEBA polarizable embedding: from ground state energies to electronic excitations. J. Chem. Theory Comput. 2016, 12, 3654–3661. 10.1021/acs.jctc.6b00385. 27340904

[ref32] Loco D.; Jurinovich S.; Cupellini L.; Menger M. F.; Mennucci B. The modeling of the absorption lineshape for embedded molecules through a polarizable QM/MM approach. Photoch. Photobio. Sci. 2018, 17, 552–560. 10.1039/C8PP00033F. 29577138

[ref33] Lipparini F.; Cappelli C.; Scalmani G.; De Mitri N.; Barone V. Analytical first and second derivatives for a fully polarizable QM/classical hamiltonian. J. Chem. Theory Comput. 2012, 8, 4270–4278. 10.1021/ct300635c. 26605590

[ref34] Lipparini F.; Cappelli C.; Barone V. Linear response theory and electronic transition energies for a fully polarizable QM/classical Hamiltonian. J. Chem. Theory Comput. 2012, 8, 4153–4165. 10.1021/ct3005062. 26605581

[ref35] Giovannini T.; Puglisi A.; Ambrosetti M.; Cappelli C. Polarizable QM/MM approach with fluctuating charges and fluctuating dipoles: the QM/FQFμ model. J. Chem. Theory Comput. 2019, 15, 2233–2245. 10.1021/acs.jctc.8b01149. 30875213

[ref36] Giovannini T.; Grazioli L.; Ambrosetti M.; Cappelli C. Calculation of ir spectra with a fully polarizable qm/mm approach based on fluctuating charges and fluctuating dipoles. J. Chem. Theory Comput. 2019, 15, 5495–5507. 10.1021/acs.jctc.9b00574. 31436976

[ref37] Giovannini T.; Riso R. R.; Ambrosetti M.; Puglisi A.; Cappelli C. Electronic transitions for a fully polarizable qm/mm approach based on fluctuating charges and fluctuating dipoles: linear and corrected linear response regimes. J. Chem. Phys. 2019, 151, 174104 10.1063/1.5121396. 31703497

[ref38] Egidi F.; Giovannini T.; Del Frate G.; Lemler P. M.; Vaccaro P. H.; Cappelli C. A combined experimental and theoretical study of optical rotatory dispersion for (R)-glycidyl methyl ether in aqueous solution. Phys. Chem. Chem. Phys. 2019, 21, 3644–3655. 10.1039/C8CP04445G. 30383044

[ref39] Steindal A. H.; Ruud K.; Frediani L.; Aidas K.; Kongsted J. Excitation energies in solution: the fully polarizable QM/MM/PCM method. J. Phys. Chem. B 2011, 115, 3027–3037. 10.1021/jp1101913. 21391548

[ref40] Schwabe T.; Olsen J. M. H.; Sneskov K.; Kongsted J.; Christiansen O. Solvation effects on electronic transitions: Exploring the performance of advanced solvent potentials in polarizable embedding calculations. J. Chem. Theory Comput. 2011, 7, 2209–2217. 10.1021/ct200258g. 26606490

[ref41] Reinholdt P.; Kongsted J.; Olsen J. M. H. Polarizable density embedding: A solution to the electron spill-out problem in multiscale modeling. J. Phys. Chem. Lett. 2017, 8, 5949–5958. 10.1021/acs.jpclett.7b02788. 29178794

[ref42] Reinholdt P.; Jørgensen F. K.; Kongsted J.; Olsen J. M. H. Polarizable density embedding for large biomolecular systems. J. Chem. Theory Comput. 2020, 16, 5999–6006. 10.1021/acs.jctc.0c00763. 32991163

[ref43] Steinmann C.; Reinholdt P.; Nørby M. S.; Kongsted J.; Olsen J. M. H. Response properties of embedded molecules through the polarizable embedding model. Int. J. Quantum Chem. 2019, 119, e25717 10.1002/qua.25717.

[ref44] Boulanger E.; Harvey J. N. QM/MM methods for free energies and photochemistry. Curr. Opin. Struct. Biol. 2018, 49, 72–76. 10.1016/j.sbi.2018.01.003. 29414514

[ref45] Giovannini T.; Egidi F.; Cappelli C. Molecular spectroscopy of aqueous solutions: a theoretical perspective. Chem. Soc. Rev. 2020, 49, 5664–5677. 10.1039/C9CS00464E. 32744278

[ref46] Ambrosetti M.; Skoko S.; Giovannini T.; Cappelli C. Quantum Mechanics/Fluctuating Charge Protocol to Compute Solvatochromic Shifts. J. Chem. Theory Comput. 2021, 17, 7146 10.1021/acs.jctc.1c00763. 34619965 PMC8582258

[ref47] Giovannini T.; Egidi F.; Cappelli C. Theory and algorithms for chiroptical properties and spectroscopies of aqueous systems. Phys. Chem. Chem. Phys. 2020, 22, 22864–22879. 10.1039/D0CP04027D. 33043930

[ref48] Giovannini T.; Lafiosca P.; Cappelli C. A general route to include Pauli repulsion and quantum dispersion effects in QM/MM approaches. J. Chem. Theory Comput. 2017, 13, 4854–4870. 10.1021/acs.jctc.7b00776. 28898079

[ref49] Kohn W.; Sham L. J. Self-consistent equations including exchange and correlation effects. Phys. Rev. 1965, 140, A1133 10.1103/PhysRev.140.A1133.

[ref50] Elstner M. SCC-DFTB: what is the proper degree of self-consistency?. J. Phys. Chem. A 2007, 111, 5614–5621. 10.1021/jp071338j. 17564420

[ref51] Foulkes W. M. C.; Haydock R. Tight-binding models and density-functional theory. Phys. Rev. B 1989, 39, 12520 10.1103/PhysRevB.39.12520. 9948117

[ref52] DFTB. https://dftb.org/parameters/download (accessed on Feb 15, 2022).

[ref53] Yang Y.; Yu H.; York D.; Cui Q.; Elstner M. Extension of the self-consistent-charge density-functional tight-binding method: third-order expansion of the density functional theory total energy and introduction of a modified effective coulomb interaction. J. Phys. Chem. A 2007, 111, 10861–10873. 10.1021/jp074167r. 17914769

[ref54] Gaus M.; Cui Q.; Elstner M. DFTB3: extension of the self-consistent-charge density-functional tight-binding method (SCC-DFTB). J. Chem. Theory Comput. 2011, 7, 931–948. 10.1021/ct100684s. PMC3509502 23204947

[ref55] Xie L.; Liu H. The treatment of solvation by a generalized Born model and a self-consistent charge-density functional theory-based tight-binding method. J. Comput. Chem. 2002, 23, 1404–1415. 10.1002/jcc.10164. 12370943

[ref56] Barone V.; Carnimeo I.; Scalmani G. Computational Spectroscopy of Large Systems in Solution: The DFTB/PCM and TD-DFTB/PCM Approach. J. Chem. Theory Comput. 2013, 9, 2052–2071. 10.1021/ct301050x. 26583552

[ref57] Cui Q.; Elstner M.; Kaxiras E.; Frauenheim T.; Karplus M. A QM/MM implementation of the self-consistent charge density functional tight binding (SCC-DFTB) method. J. Phys. Chem. B 2001, 105, 569–585. 10.1021/jp0029109. 12632471

[ref58] Nottoli M.; Lipparini F. General formulation of polarizable embedding models and of their coupling. J. Chem. Phys. 2020, 153, 224108 10.1063/5.0035165. 33317291

[ref59] Gómez S.; Giovannini T.; Cappelli C. Absorption spectra of xanthines in aqueous solution: A computational study. Phys. Chem. Chem. Phys. 2020, 22, 5929–5941. 10.1039/C9CP05420K. 32115599

[ref60] Puglisi A.; Giovannini T.; Antonov L.; Cappelli C. Interplay between conformational and solvent effects in UV-visible absorption spectra: Curcumin tautomers as a case study. Phys. Chem. Chem. Phys. 2019, 21, 15504–15514. 10.1039/C9CP00907H. 31259324

[ref61] Skoko S.; Ambrosetti M.; Giovannini T.; Cappelli C. Simulating Absorption Spectra of Flavonoids in Aqueous Solution: A Polarizable QM/MM Study. Molecules 2020, 25, 5853 10.3390/molecules25245853. PMC7764712 33322361

[ref62] Gómez S.; Egidi F.; Puglisi A.; Giovannini T.; Rossi B.; Cappelli C. Unlocking the power of resonance Raman spectroscopy: The case of amides in aqueous solution. J. Mol. Liq. 2022, 346, 117841 10.1016/j.molliq.2021.117841.

[ref63] Gómez S.; Rojas-Valencia N.; Giovannini T.; Restrepo A.; Cappelli C. Ring Vibrations to Sense Anionic Ibuprofen in Aqueous Solution as Revealed by Resonance Raman. Molecules 2022, 27, 442 10.3390/molecules27020442. 35056755 PMC8780161

[ref64] Baerends E.; DFTB, version 2020.x; Theoretical Chemistry, Vrije Universiteit: Amsterdam, The Netherlands, 2020. http://www.scm.com (accessed on Feb 15, 2022).

[ref65] Olszówka M.; Russo R.; Mancini G.; Cappelli C. A computational approach to the resonance Raman spectrum of doxorubicin in aqueous solution. Theor. Chem. Acc. 2016, 135, 27 10.1007/s00214-015-1781-9.

[ref66] Brancolini G.; Kokh D. B.; Calzolai L.; Wade R. C.; Corni S. Docking of Ubiquitin to Gold Nanoparticles. ACS Nano 2012, 6, 9863–9878. 10.1021/nn303444b. 23033917

[ref67] Jawad B.; Poudel L.; Podgornik R.; Steinmetz N. F.; Ching W.-Y. Molecular mechanism and binding free energy of doxorubicin intercalation in DNA. Phys. Chem. Chem. Phys. 2019, 21, 3877–3893. 10.1039/C8CP06776G. 30702122

[ref68] Hillig K. W.; Morris M. D. Pre-resonance Raman spectra of adriamycin. Biochem. Biophys. Res. Commun. 1976, 71, 1228–1233. 10.1016/0006-291X(76)90785-3. 971308

[ref69] Bellina B.; Compagnon I.; Joly L.; Albrieux F.; Allouche A.-R.; Bertorelle F.; Lemoine J.; Antoine R.; Dugourd P. UV spectroscopy of entire proteins in the gas phase. Int. J. Mass Spectrom. 2010, 297, 36–40. 10.1016/j.ijms.2010.05.015.

[ref70] Spiegelman F.; Tarrat N.; Cuny J.; Dontot L.; Posenitskiy E.; Martí C.; Simon A.; Rapacioli M. Density-functional tight-binding: basic concepts and applications to molecules and clusters. Adv. Phys. X 2020, 5, 1710252 10.1080/23746149.2019.1710252. 33154977 PMC7116320

[ref71] Elstner M.; Porezag D.; Jungnickel G.; Elsner J.; Haugk M.; Frauenheim T.; Suhai S.; Seifert G. Self-consistent-charge density-functional tight-binding method for simulations of complex materials properties. Phys. Rev. B 1998, 58, 7260–7268. 10.1103/PhysRevB.58.7260.

[ref72] Gaus M.; Goez A.; Elstner M. Parametrization and Benchmark of DFTB3 for Organic Molecules. J. Chem. Theory Comput. 2013, 9, 338–354. 10.1021/ct300849w. 26589037

[ref73] Rick S. W.; Stuart S. J.; Berne B. J. Dynamical fluctuating charge force fields: Application to liquid water. J. Chem. Phys. 1994, 101, 6141–6156. 10.1063/1.468398.

[ref74] Lown J. W. Discovery and development of anthracycline antitumour antibiotics. Chem. Soc. Rev. 1993, 22, 165–176. 10.1039/cs9932200165.

[ref75] Chaires J. B.; Satyanarayana S.; Suh D.; Fokt I.; Przewloka T.; Priebe W. Parsing the Free Energy of Anthracycline Antibiotic Binding to DNA. Biochemistry (Mosc.) 1996, 35, 2047–2053. 10.1021/bi952812r. 8652545

[ref76] Gewirtz D. A critical evaluation of the mechanisms of action proposed for the antitumor effects of the anthracycline antibiotics adriamycin and daunorubicin. Biochem. Pharmacol. 1999, 57, 727–741. 10.1016/S0006-2952(98)00307-4. 10075079

[ref77] Barone G.; Guerra C. F.; Gambino N.; Silvestri A.; Lauria A.; Almerico A. M.; Bickelhaupt F. M. Intercalation of Daunomycin into Stacked DNA Base Pairs. DFT Study of an Anticancer Drug. J. Biomol. Struct. Dyn. 2008, 26, 115–129. 10.1080/07391102.2008.10507229. 18533732

[ref78] Poudel L.; Wen A. M.; French R. H.; Parsegian V. A.; Podgornik R.; Steinmetz N. F.; Ching W.-Y. Electronic structure and partial charge distribution of doxorubicin in different molecular environments. ChemPhysChem 2015, 16, 1451–1460. 10.1002/cphc.201402893. 25728554

[ref79] Zhu S.; Yan L.; Ji X.; Lu W. Conformational diversity of anthracycline anticancer antibiotics: A density functional theory calculation. J. Mol. Struct. THEOCHEM 2010, 951, 60–68. 10.1016/j.theochem.2010.04.008.

[ref80] Egidi F.; Lo Gerfo G.; Macchiagodena M.; Cappelli C. On the nature of charge-transfer excitations for molecules in aqueous solution: a polarizable QM/MM study. Theor. Chem. Acc. 2018, 137, 1–12. 10.1007/s00214-018-2259-3.

[ref81] Giovannini T.; Macchiagodena M.; Ambrosetti M.; Puglisi A.; Lafiosca P.; Lo Gerfo G.; Egidi F.; Cappelli C. Simulating vertical excitation energies of solvated dyes: From continuum to polarizable discrete modeling. Int. J. Quantum Chem. 2019, 119, e25684 10.1002/qua.25684.

[ref82] Jawad B.; Poudel L.; Podgornik R.; Ching W.-Y. Thermodynamic Dissection of the Intercalation Binding Process of Doxorubicin to dsDNA with Implications of Ionic and Solvent Effects. J. Phys. Chem. B 2020, 124, 7803–7818. 10.1021/acs.jpcb.0c05840. 32786213

[ref83] Jia M.; Song X.; Zhang Q.; Yang D. A Theoretical Investigation About the Excited State Dynamical Mechanism for Doxorubicin Sensor. J. Cluster Sci. 2018, 29, 673–678. 10.1007/s10876-018-1388-0.

[ref84] Manfait M.; Bernard L.; Theophanides T. Resonance and pre-resonance Raman spectra of the antitumor drugs adriamycin and daunomycin. J. Raman Spectrosc. 1981, 11, 68–74. 10.1002/jrs.1250110205.

[ref85] Manfait M.; Alix A. J.; Jeannesson P.; Jardillier J.-C.; Theophanides T. Interaction of adriamycin with DNA as studied by resonance Raman spectroscopy. Nucleic Acids Res. 1982, 10, 3803–3816. 10.1093/nar/10.12.3803. 7111023 PMC320754

[ref86] Angeloni L.; Smulevich G.; Marzocchi M. Absorption, fluorescence and resonance Raman spectra of adriamycin and its complex with DNA. Spectrochim. Acta A-M 1982, 38, 213–217. 10.1016/0584-8539(82)80199-2.

[ref87] Smulevich G.; Mantini A. R.; Feis A.; Marzocchi M. P. Resonance Raman spectra and transform analysis of anthracyclines and their complexes with DNA. J. Raman Spectrosc. 2001, 32, 565–578. 10.1002/jrs.721.

[ref88] Lee C.-J.; Kang J.-S.; Kim M.-S.; Lee K.-P.; Lee M.-S. The study of doxorubicin and its complex with DNA by SERS and UV-resonance Raman spectroscopy. Bull. Korean Chem. Soc. 2004, 25, 1211–1216. 10.5012/bkcs.2004.25.8.1211.

[ref89] Yan Q.; Priebe W.; Chaires J. B.; Czernuszewicz R. S. Interaction of doxorubicin and its derivatives with DNA: Elucidation by resonance Raman and surface-enhanced resonance Raman spectroscopy. Biospectroscopy 1997, 3, 307–316. 10.1002/(SICI)1520-6343(1997)3:4<307::AID-BSPY6>3.0.CO;2-0.

[ref90] Florêncio e Silva E.; Machado E. S.; Vasconcelos I. B.; Junior S. A.; Dutra J. D. L.; Freire R. O.; da Costa N. B. Are the Absorption Spectra of Doxorubicin Properly Described by Considering Different Tautomers?. J. Chem. Inf. Model. 2020, 60, 513–521. 10.1021/acs.jcim.9b00785. 31833765

[ref91] Pickart C. M.; Eddins M. J. Ubiquitin: structures, functions, mechanisms. BBA - Mol. Cell. Res. 2004, 1695, 55–72. 10.1016/j.bbamcr.2004.09.019. 15571809

[ref92] Glickman M. H.; Ciechanover A. The Ubiquitin-Proteasome Proteolytic Pathway: Destruction for the Sake of Construction. Physiol. Rev. 2002, 82, 373–428. 10.1152/physrev.00027.2001. 11917093

[ref93] Schnell J. D.; Hicke L. Non-traditional Functions of Ubiquitin and Ubiquitin-binding Proteins. J. Biol. Chem. 2003, 278, 35857–35860. 10.1074/jbc.R300018200. 12860974

[ref94] Mukhopadhyay D.; Riezman H. Proteasome-Independent Functions of Ubiquitin in Endocytosis and Signaling. Science 2007, 315, 201–205. 10.1126/science.1127085. 17218518

[ref95] Goldstein G.; Scheid M.; Hammerling U.; Schlesinger D. H.; Niall H. D.; Boyse E. A. Isolation of a polypeptide that has lymphocyte-differentiating properties and is probably represented universally in living cells. P. Natl. Acad. Sci. USA 1975, 72, 11–15. 10.1073/pnas.72.1.11. PMC432229 1078892

[ref96] Antoine R.; Dugourd P. Visible and ultraviolet spectroscopy of gas phase protein ions. Phys. Chem. Chem. Phys. 2011, 13, 16494–16509. 10.1039/c1cp21531k. 21811728

[ref97] Creed D. The photophysics and photochemistry of the near-UV absorbing amino acids-II. Tyrosyne and its simple derivatives. Photochem. Photobiol. 1984, 39, 563–575. 10.1111/j.1751-1097.1984.tb03891.x.

[ref98] Fornander L. H.; Feng B.; Beke-Somfai T.; Nordén B. UV Transition Moments of Tyrosine. J. Phys. Chem. B 2014, 118, 9247–9257. 10.1021/jp5065352. 25020040

[ref99] Del Galdo S.; Mancini G.; Daidone I.; Zanetti Polzi L.; Amadei A.; Barone V. Tyrosine absorption spectroscopy: Backbone protonation effects on the side chain electronic properties. J. Comput. Chem. 2018, 39, 1747–1756. 10.1002/jcc.25351. 29756218

[ref100] Weber G. Fluorescence-polarization spectrum and electronic-energy transfer in tyrosine, tryptophan and related compounds. Biochem. J. 1960, 75, 335 10.1042/bj0750335. 13843297 PMC1204430

[ref101] Longworth J. Excited States of Proteins and Nucleic Acids; Plenum Press: New York, 1971; pp 319–484.

[ref102] Nishimoto Y. Time-dependent density-functional tight-binding method with the third-order expansion of electron density. J. Chem. Phys. 2015, 143, 094108 10.1063/1.4929926. 26342360

[ref103] Vijay-Kumar S.; Bugg C. E.; Cook W. J. Structure of ubiquitin refined at 1.8Å resolution. J. Mol. Biol. 1987, 194, 531–544. 10.1016/0022-2836(87)90679-6. 3041007

[ref104] Wyer J. A.; Ehlerding A.; Zettergren H.; Kirketerp M.-B. S.; Brøndsted Nielsen S. Tagging of Protonated Ala-Tyr and Tyr-Ala by Crown Ether Prevents Direct Hydrogen Loss and Proton Mobility after Photoexcitation: Importance for Gas-Phase Absorption Spectra, Dissociation Lifetimes, and Channels. J. Phys. Chem. A 2009, 113, 9277–9285. 10.1021/jp904053d. 19639974

[ref105] Cappelli C. Unpublished.

[ref106] Giovannini T.; Lafiosca P.; Chandramouli B.; Barone V.; Cappelli C. Effective yet reliable computation of hyperfine coupling constants in solution by a QM/MM approach: Interplay between electrostatics and non-electrostatic effects. J. Chem. Phys. 2019, 150, 124102 10.1063/1.5080810. 30927869

[ref107] Giovannini T.; Ambrosetti M.; Cappelli C. Quantum confinement effects on solvatochromic shifts of molecular solutes. J. Phys. Chem. Lett. 2019, 10, 5823–5829. 10.1021/acs.jpclett.9b02318. 31518133

