# Supplementary material for: Absorption Properties of Large Complex Molecular Systems: The DFTB/Fluctuating Charge Approach
Source: J Chem Theory Comput. 2022 Feb 20;18(3):1765–79. doi: 10.1021/acs.jctc.1c01066 (PMC8908768; doi:10.1021/acs.jctc.1c01066)
Supplement: Supplementary file 1 — ct1c01066_si_001.pdf [file ct1c01066_si_001.pdf]

Supporting Information: Absorption Properties of Large Complex Molecular Systems: The DFTB/Fluctuating Charge Approach

Piero Lafiosca, Sara Gómez, Tommaso Giovannini,* and Chiara Cappelli*

Scuola Normale Superiore, Classe di Scienze, Piazza dei Cavalieri 7, 56126, Pisa, Italy

E-mail: tommaso.giovannini@sns.it; chiara.cappelli@sns.it

S1 Computational details

Table S1: Technical settings for TD-DFTB calculations: number of computed excited states; minimum value of oscillator strength for single orbital transitions; maximum energy of single orbital transitions.

System	Nr. excited states	Min. osc. str.	Max. trans. energy (eV)
DOX/water	100	-	-
DOX/DNA/water	300	-	-
UBI/water	400	0.0005	6.199

S2 Doxorubicin

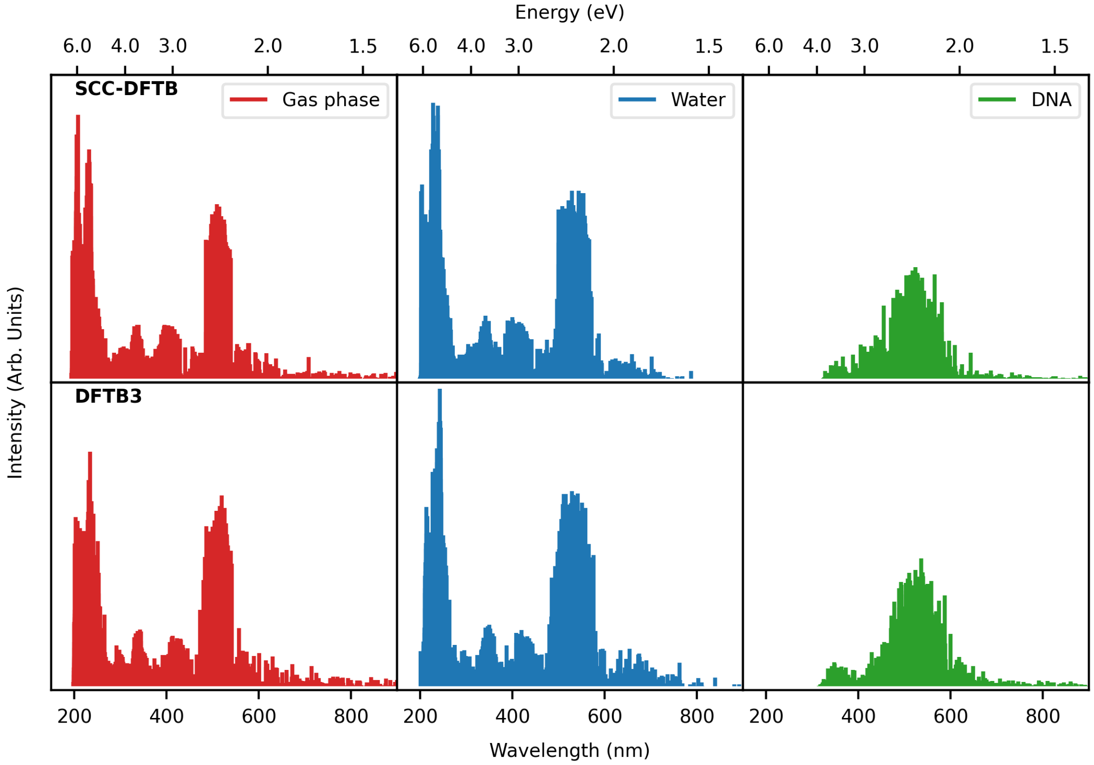

Figure S1: Stick spectra (data are not normalized) of doxorubicin *in vacuo* (red line), in water (blue line) and intercalated in DNA (green line), as obtained with two different DFTB Hamiltonians.

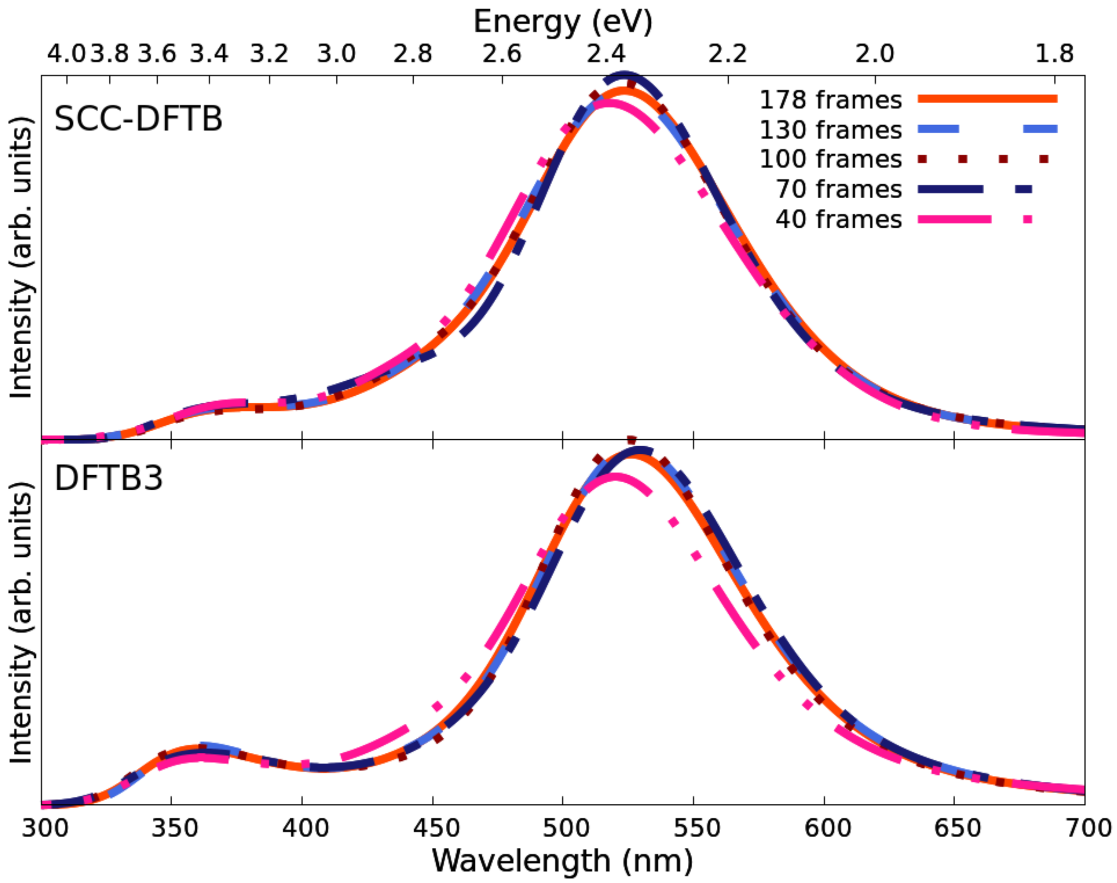

Figure S2: Convergence test for absorption spectra of the DOX/Water/DNA system.

Table S2: Vertical excitation energy (VEE) of doxorubicin in gas phase and in aqueous solution. The gas-phase result indicates the VEEs obtained by averaging the results for three most stable doxorubicin conformers. In parenthesis, the gas-phase VEEs obtained by removing the water molecules from the MD snapshots, i.e. the data discussed in the main text, are reported.

Level of theory	VEE (gas phase, eV)	VEE (water, eV)	Shift (eV)
CAM-B3LYP/6-31+G*	3.10	2.91	0.19
SCC-DFTB	2.46 (2.41)	2.33	0.13 (0.08)
DFTB3	2.44 (2.43)	2.35	0.11 (0.08)

S3 Ubiquitin

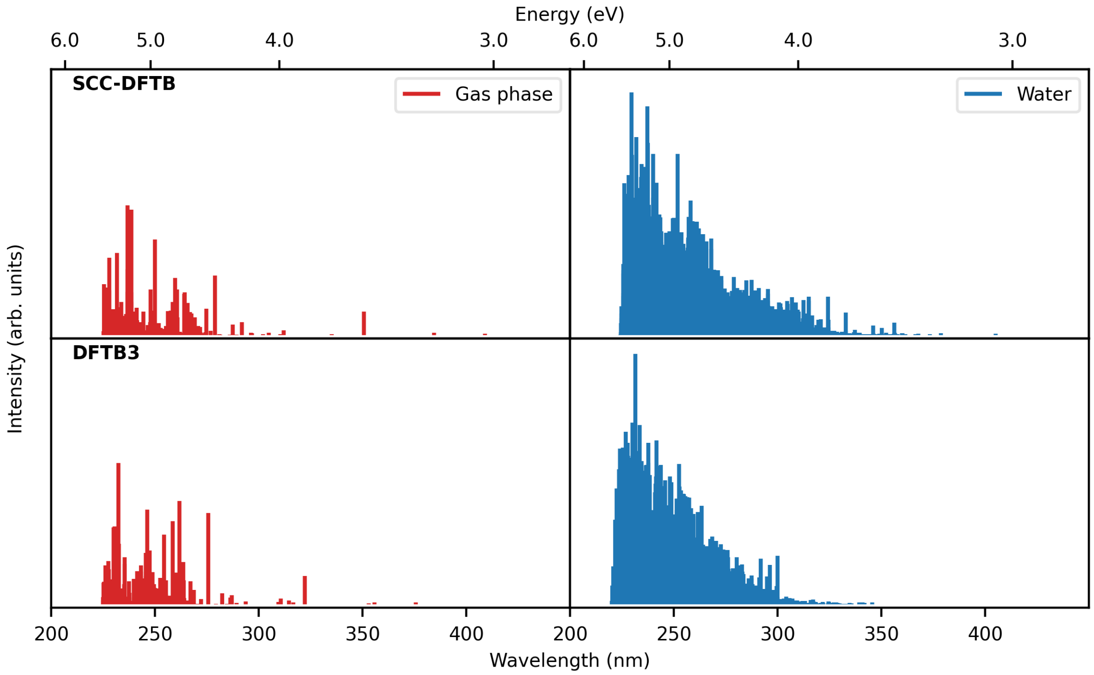

Figure S3: Stick spectra (data are not normalized) of ubiquitin *in vacuo* (red line) and in aqueous solution (blue line), as obtained with two DFTB Hamiltonians.

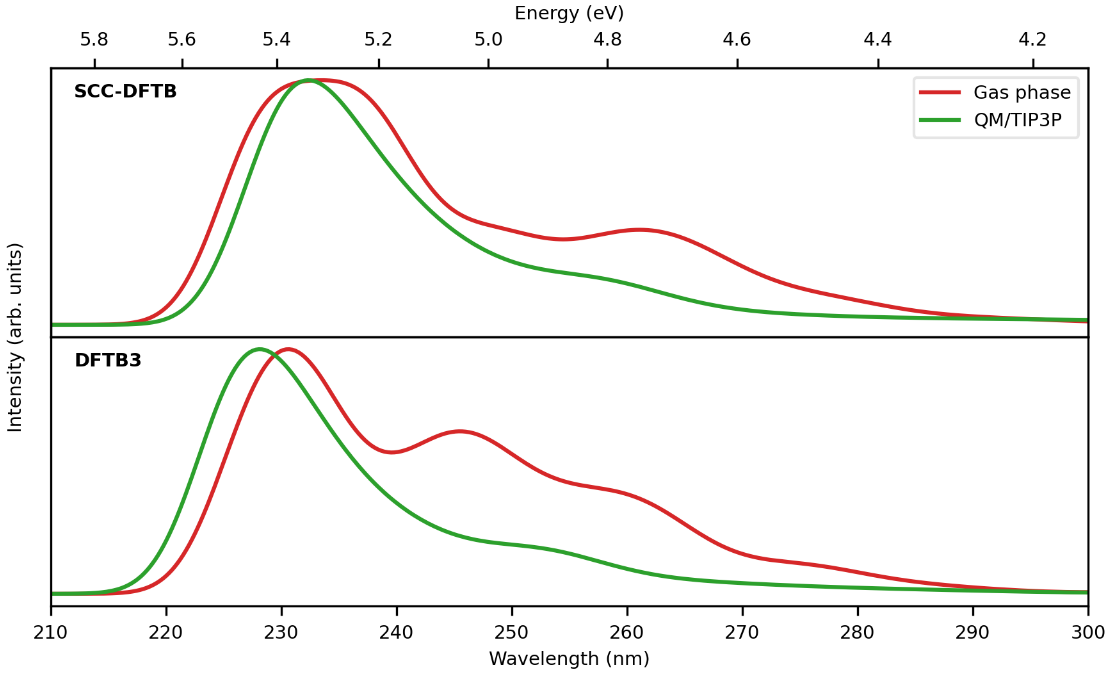

Figure S4: Absorption spectra of ubiquitin (modelled at the SCC or DFTB3 levels) in aqueous solution, where water molecules are treated with the non-polarizable TIP3P force-field.
